# Sex‐dependent molecular landscape of Alzheimer's disease revealed by large‐scale single‐cell transcriptomics

**DOI:** 10.1002/alz.14476

**Published:** 2024-12-31

**Authors:** Mohamed Soudy, Sophie Le Bars, Enrico Glaab

**Affiliations:** ^1^ Biomedical Data Science Group Luxembourg Centre for Systems Biomedicine (LCSB) University of Luxembourg Esch‐sur‐Alzette Luxembourg

**Keywords:** Alzheimer's disease, apoptosis, cell death, network analysis, pathway analysis, sex differences, single‐cell sequencing, transcriptome analysis

## Abstract

**INTRODUCTION:**

Alzheimer's disease (AD) shows significant sex differences in prevalence and clinical manifestations, but the underlying molecular mechanisms remain unclear.

**METHODS:**

This study used a large‐scale, single‐cell transcriptomic atlas of the human prefrontal cortex to investigate sex‐dependent molecular changes in AD. Our approach combined cell type–specific and sex‐specific differential gene expression analysis, pathway enrichment, gene regulatory network construction, and cell–cell communication analysis to identify sex‐dependent changes.

**RESULTS:**

We found significant sex‐specific gene expression patterns and pathway alterations in AD. Male astrocytes showed changes in cell death pathways, with *RPTOR* as a key regulator, while female astrocytes had alterations in Wnt signaling and cell cycle regulation. Cell–cell communication analysis uncovered sex‐specific intercellular signaling patterns, with male‐specific impacts on apoptosis‐related signaling and female‐specific alterations in Wnt and calcium signaling.

**DISCUSSION:**

This study reveals sex‐dependent gene expression patterns, pathway alterations, and intercellular communication changes, suggesting the need for sex‐specific approaches in AD research.

**Highlights:**

- Single‐cell transcriptomics reveals significant sex‐specific molecular signatures in Alzheimer's disease (AD).
- Male astrocytes show enhanced modulation of apoptotic and cell death pathways in AD; female astrocytes exhibit distinct alterations in Wnt signaling and cell‐cycle regulation.
- Sex‐dimorphic changes in mitochondrial gene expression are observed in excitatory neurons, suggesting divergent energy metabolism profiles may contribute to AD sex differences.
- *RPTOR* is identified as a key regulator of male‐specific changes in astrocytes, implicating the mechanistic target of rapamycin pathway in sex‐dependent AD pathology.
- New cell–cell communication analyses reveal sex‐specific patterns of intercellular signaling, providing insights into how cellular interactions may differentially contribute to AD pathology in males and females.

## 1 BACKGROUND

Alzheimer's disease (AD) is characterized by strong heterogeneity in prevalence, progression, and manifestation between the sexes, with women bearing a disproportionate burden of the disease.^1^, ^2^ Accumulating evidence from epidemiologic, clinical, and biological studies underscores the importance of understanding these sex differences in AD and their potential implications for diagnosis and treatment.^3^, ^4^

Women not only have a higher lifetime risk of developing AD, but also experience more rapid cognitive decline and greater brain atrophy once diagnosed.^5^ However, the extent of impairment can vary significantly depending on which specific symptoms, cognitive domains, and comorbidities are considered.^6^, ^7^ For instance, females tend to show greater impairment in verbal memory and faster cognitive decline,^8^ while males often exhibit more pronounced behavioral symptoms such as apathy, wandering, abusiveness, and social impropriety.^9^ These differential patterns of impairment underscore the complexity of sex differences in AD and the importance of tailored approaches in both research and clinical practice.

Several factors contribute to the observed sex differences in AD. Longevity plays a role, as women tend to live longer than men, increasing their likelihood of developing age‐related neurodegenerative diseases.^10^ However, this alone does not fully explain the disparity. Hormonal influences, particularly the postmenopausal decline in estrogen, have been implicated in increased AD risk and progression in women.^11^ In addition, genetic factors such as the apolipoprotein E (*APOE*) ε4 allele confer a greater risk of AD in women compared to men.^12^

Despite these known differences, the molecular basis of sex‐specific AD pathology remains poorly understood, particularly at the cellular level. Most studies to date have focused on bulk tissue analyses, which may obscure cell type–specific changes and fail to capture the heterogeneity of cellular responses in the aging and diseased brain.^13^ Single‐cell transcriptomics offers a new approach to address this knowledge gap by providing high‐resolution insights into cell type–specific gene expression patterns and regulatory networks.^14^

Recent technological advances have enabled the generation of large‐scale single‐cell datasets, providing unprecedented opportunities to explore the cellular and molecular landscapes of complex diseases such as AD.^15^ The comprehensive single‐cell transcriptome atlas of the aged human prefrontal cortex, comprising 2.3 million cells from 427 individuals with varying degrees of AD pathology and cognitive impairment, represents a valuable resource for investigating sex‐specific changes in AD at cellular resolution.^4^

Previous analyses of this dataset have revealed novel patterns and relationships within the cellular landscape of the aged prefrontal cortex, identifying distinct cellular subpopulations and gene expression profiles that correlate with AD pathology and cognitive decline.^16^, ^17^ However, these studies have not specifically examined sex‐specific changes between AD and healthy groups using detailed statistical and mechanistic pathway and network analyses, representing a critical gap in our understanding of AD pathology.^18^

To address this knowledge gap, our study exploits this comprehensive single‐cell transcriptome atlas to investigate sex‐related dysregulations in AD at unprecedented resolution and scale. By focusing on unexplored aspects of sex‐specific gene expression patterns and cellular processes, we aim to gain detailed insights into how AD manifests differently between the sexes.

Our study uses a novel combination of advanced bioinformatics approaches to better understand the complexity of sex‐specific AD pathology. In addition to accounting for sex dependencies in differential gene expression analysis to identify sex‐related transcriptional changes in different cell types, we conduct pathway analyses and gene regulatory network (GRN) analyses. This is done to obtain a systems‐level view of the molecular interactions underlying sex differences in AD.

A key novelty in our methodology is the integration of cell–cell communication analysis, which allows us to examine how sex‐specific changes in one cell type may affect other cell types in the AD brain. This approach provides a more comprehensive understanding of the complex cellular interactions that contribute to AD pathology and how these may differ between the sexes.

Overall, the insights gained from studying sex differences in AD at the single‐cell level have the potential to significantly advance our understanding of AD pathogenesis and progression.

## 2 METHODS

### 2.1 Quality control and sample preprocessing

Single‐cell RNA sequencing (scRNA‐seq) samples were derived from a comprehensive single‐cell transcriptomic atlas of the aged human prefrontal cortex.^4^ This dataset includes 2.3 million cells, obtained from *post mortem* brain samples of 427 individuals with different types of dementia and cognitive impairment, of which the subset of 306 samples with either confirmed AD status or healthy control (HC) status was included. Varying levels of cognitive impairment and different stages of AD pathology are covered in this data (see overview in Table 1).

**TABLE 1:** Summary of study groups included in the cohort.

Group	Number of individuals	Braak stages	Description
Controls	146 (72 M; 74 F)	N.A.	Controls with no AD or other known brain disorders
Early AD	19 (14 M; 5 F)	0, I, II	Early stages of AD
Intermediate AD	59 (37 M; 22 F)	III, IV	Moderate progression of AD
Late AD	82 (28 M; 54 F)	V, VI	Advanced stages of AD

*Note*: This table provides an overview of study participants categorized into three groups based on the Braak staging system, which reflects the severity of AD. The groups include Controls and Early‐AD (Braak stages 0–II), Intermediate AD (Braak stages III–IV), and Late AD (Braak stages V–VI), indicating the progression from no or early AD to advanced stages. Sample sizes are provided for each group with sex‐stratified counts.

Abbreviations: AD, Alzheimer's disease; F, female; M, male.

### 2.2 Cell type annotation

The software SCANPY^19^ was used to process and cluster the expression profiles, and the data integration workflow of the Seurat package (version 5.1.0, RRID:SCR_016341) using the SCTransform normalization was applied to align data from individual batches.^20^ High‐resolution cell clusters were identified using the Seurat functions FindNeighbors and FindClusters, which use the Leiden clustering algorithm.^21^ Cell types were annotated using previously published marker genes and scRNA‐seq data from public resources, including the Allen Institute's Cell Types Database.^4^, ^22^ Spearman rank correlation coefficients were computed to compare the average expression profiles of previously defined neuronal subpopulations to those identified in the source publication for the single‐cell transcriptomic atlas.

**RESEARCH IN CONTEXT**

1. **Systematic review**: The authors analyzed a large‐scale single‐cell transcriptomic dataset of the human prefrontal cortex and reviewed the biomedical literature to contextualize findings on sex differences in Alzheimer's disease (AD) pathology.
2. **Interpretation**: Our study reveals extensive sex‐dependent molecular changes in AD across multiple cell types, providing new insights into sex‐specific gene expression, pathways, and regulatory networks at single‐cell resolution.
3. **Future directions**: This article provides a comprehensive framework for investigating sex‐specific aspects of AD pathology. Building on these findings, future research should focus on: (a) functional validation of key sex‐specific genes and pathways; (b) temporal dynamics of sex‐specific changes in AD progression; (c) identification of sex‐specific AD biomarkers; and (d) tailoring therapeutic interventions to patient sex, where appropriate.

### 2.3 Transcriptomics differential expression analysis

We conducted differential gene expression analyses for four cell types with established involvement in AD, including excitatory and inhibitory neurons, oligodendrocytes, immune cells, and astrocytes. Specifically, the Poisson generalized linear model implemented in the FindMarkers function of the Seurat package^20^ was used to identify differentially expressed genes (DEGs) between AD patients and HCs. *P* values were corrected for multiple hypothesis testing by applying the Benjamini–Hochberg approach.^23^ If the adjusted *P* value for a gene was < 0.05 and a minimum effect size (abs[logFC] > 0.25) was observed, the corresponding gene was defined as significantly differentially expressed. Using this procedure, significant DEGs were identified and assigned to the following categories:

1. Sex‐neutral DEGs: Genes with significant expression changes in the same direction for both females and males (adjusted *P* value < 0.05 in each sex).
2. Sex‐specific DEGs: Genes showing significant differential expression in only one sex (adjusted *P* value < 0.05) while remaining well below the threshold of significance in the other (nominal *p* value > 0.1). This stringent criterion for non‐significance helps avoid misclassification due to random variation around the conventional 0.05 threshold. Using an interaction term analysis as an alternative approach would require greater statistical power and, if the variance of log fold changes is high, may result in an increased rate of false positive discoveries.
3. Sex‐dimorphic DEGs: Genes demonstrating significant expression changes in both sexes, but in opposite directions (false discovery rate [FDR] < 0.05 for both females and males and opposite sign of the log fold change).

### 2.4 Enrichment analysis

We performed gene set enrichment analyses using the R package clusterProfiler (version 4.12.0 RRID: SCR_016884) to evaluate global patterns of sex‐specific alterations in cellular processes, deriving pathway annotation data from the publicly available databases Gene Ontology (GO; RRID: SCR_010326)^24^ and Kyoto Encyclopedia of Genes and Genomes (KEGG; RRID: SCR_018145). Human gene annotations were extracted from the R package org*.Hs.eg.db* (version 3.19.1). An adjusted *P* value < 0.05 was used as a threshold to determine significance between AD and controls for each sex, with a specificity threshold > 0.1 of the nominal *P* value for the other sex to identify processes showing a robust sex‐specific alteration (i.e., sex‐specific significance requires an adjusted *P* value < 0.05 in one sex, and nominal *p* > 0.1 in the other sex). Finally, the enrichment analysis results were visualized using the R package enrichplot (version 1.24.0).^25^

### 2.5 Cell–cell communication analysis

We investigated AD‐associated alterations in cell–cell communication using the software NicheNetR (version 2.1.5) and CellChatDB (version 1.6.1) to determine ligand activities and hub genes involved in altered communication events.^26^, ^27^ The expression data for sender and receiver cells were filtered to identify the subset of genes expressed in > 10% of the cells, which were then used to predict ligand activities and determine the most relevant ligands. Next, to identify the pathways most strongly affected by alterations in cell–cell communication events, enrichment analyses were conducted using the DEGs intersected with the identified top ligands and top receptors expressed in the relevant cell types (using a significance threshold of *P* < 0.05). These pathway enrichment analyses were performed using the clusterProfiler software and annotations from the GO and KEGG database.

### 2.6 Network analysis

GRNs were built for the DEGs identified in the statistical analyses for each of the four cell types of interest. Regulatory interactions between the DEGs for each cell type were retrieved from the Mammalian ResNet database using the MetaCore web portal (RRID: SCR_008125). Only human interactions annotated for brain tissues were considered. Moreover, interactions were filtered to include only the following mechanism annotations: binding, transcription regulation, influence on expression, co‐regulation of transcription, and regulation.

Phenotype‐specific GRNs for AD and control conditions were then built using a dedicated network construction technique.^28^ In short, this method applies a genetic algorithm to filter and select interactions such that they align with the Booleanized gene expression states of the disease and control phenotypes. Because certain interactions obtained from the ResNet database lack the information on their activating or inhibiting effect, the software uses the available gene expression data and network topological information to deduce missing regulatory effect information.^28^

### 2.7 Proteomics data analysis and integration

To validate our transcriptomics findings at the protein level, we analyzed a previously published tandem mass tag mass spectrometry proteomics dataset from an AD case–control study.^28^ The normalized proteomics data and associated metadata were downloaded from the Synapse archive of the original study (ID: syn25006657). Our analysis focused on the subset of samples from the prefrontal cortex, covering 123 AD patients and 168 control samples.

For the differential expression analysis of the proteomics data, we performed linear regression modeling using the software package limma (v3.60.4)^29^ to compare AD versus control groups separately within each sex, accounting for age at death as a covariate. Multiple testing corrections were applied using the Benjamini–Hochberg method.^23^ This approach uses empirical Bayes moderation of standard errors, providing a more robust statistical framework than traditional *t* tests for differential expression analysis. Finally, we categorized protein expression changes as sex specific, sex dimorphic, or sex neutral, following the same definitions used for the transcriptomics data (see section 2.3).

To integrate the proteomics and transcriptomics results, we conducted an intersection analysis between the sex‐dependent differentially expressed proteins (DEPs) and the cell type‐specific DEGs. Specifically, for each cell type, we computed the overlap between (1) male‐specific DEGs and male‐specific DEPs, (2) female‐specific DEGs and female‐specific DEPs, and (3) sex‐dimorphic DEGs and sex‐dimorphic DEPs.

## 3 RESULTS

### 3.1 Differential gene expression

Using the cell‐type annotations from the source publication^4^ and analyzing each cell type by determining sex‐dependent differential gene expression between AD patients and control subjects separately within each sex, as described in the Methods section, we identified several statistically significant alterations after multiple hypothesis testing adjustments. The top‐ranked DEGs (up to 10) with male‐specific, female‐specific, and sex‐dimorphic alterations are shown in Tables 2, 3, 4, respectively. The complete rankings of DEGs for the different cell type clusters are provided Table S1 in supporting information, and Table S2 in supporting information provides the numbers of identified sex‐dependent DEGs for each cell type. In the following, mentions of increased or decreased male‐ or female‐specific expression refer to significant overexpression in AD patients compared to controls within the respective sex (FDR < 0.05), with no indication of a significant change in AD patients relative to controls in the opposite sex, even at the nominal level (*p* > 0.1).

**TABLE 2:** Male‐specific DEGs in each cell type with the highest absolute log fold change between AD and controls (FDR  <  0.05, up to 10 genes per cell type shown), that is, representing the DEGs found to be significant in only one sex (FDR  <  0.05), and which do not approach significance in the other sex (*p*  >  0.1).

Cell type	Gene symbol	Min and max FDR significance
Astrocytes	*LMCD1‐AS1, PCSK5, PIK3C2A, MGAT5, ERBB4, LYST, HIBCH, DDX58*	3.19e‐30–0.003
Immune cells	*SPON1, AC015712.1, AP000547.3, AL356417.3, RPS13, MECOM, RPL30, RPL32*	1.97e‐42–0.018
Inhibitory neurons	*EGR1, MUC20, NOX3, NLRP2, LRRIQ1, AL109615.3, AL117329.1,AC018742.1*	3.88e17–0.033
Oligodendrocytes	*FNDC3A, ANKRD44, ZFAND3, FABP5, CHD1, GNG7, VMP1*	3.35e‐23–6.88e‐11
Vasculature cells	*ALDH1L1, FZD9, RNF41, ADGRE5, SLC26A7, COL9A3, AFF2, GRTP1*	3.83e‐30–0.0434
OPCs	*RSPO4, REXO5, PFKP, LINC01515, COL5A3*	1.85e‐95–9.67e‐05
Excitatory neurons set 1	*SYAP1, STRBP, TENM1, ESR1, CPOX, PLCH1*	1.10e‐52–0.023
Excitatory neurons set 2	*LTBP1, MYO1E, MYBPC1, ABCC3, NLGN4Y, CXCL13*	3.47e‐77–0.015
Excitatory neurons set 3	*LIMA1, RPL31, ADAMTS18, PAX6, COL5A2, AC020911.2, LAMB3, USP9Y*	1.87e‐14–5.77e‐13

*Note*: Genes with increased expression in male AD patients are depicted in red, while underexpressed genes are represented in blue.

Abbreviations: AD, Alzheimer's disease; DEGs, differentially expressed genes; FDR, false discovery rate; max, maximum; min, minimum; OPCs, oligodendrocyte precursor cells.

**TABLE 3:** Female‐specific DEGs in each cell type with the highest absolute log fold change between AD and controls (FDR  <  0.05, up to 10 genes per cell type shown), that is, representing the DEGs found to be significant in only one sex (FDR  <  0.05), and which do not approach significance in the other sex (*p*  >  0.1).

Cell type	Gene symbol	Min and max FDR significance
Astrocytes	*BICC1, CCL2, PLEKHG1, MCC, AC018616.1, NCAM2, PDZRN3, DNER*	3.23e‐10–0.0001
Immune cells	*AC069277.1, USP18, HELZ2, HS3ST2, VMO1, TENM3, RAB3C, AC104041.1, GALNT13*	4.31e‐21–0.028
Inhibitory neurons	*INHBA, MT2A, APOD, TMEM233, TLL1, SMCR5, ISG20*	2.23e‐64–0.022
Oligodendrocytes	*DNAJB1, HSP90B1, ZBTB7C, FAM198B, GPR158, ALAS1, PLAUR, GDNF*	2.18e‐42–0.001
Vasculature cells	*CRIP1, PPP1R3C, SUSD5, COL8A1, COL15A1, CNTN6, FGF1, ABCA9‐AS1*	1.19e‐31–0.037
OPCs	*APOA1‐AS, GABRB2, LOXL2, RAB39A*	2.74e‐17–0.001
Excitatory neurons set 1	*TUBB2A, PIK3C2G, TAGLN3, ENPP1, KIF14, ZNF462*	2.20e‐21–9.87e‐24
Excitatory neurons set 2	*OSBPL1A, KCNH1, KMO, DDX60L, AL132780.1, PIP4K2A, KDM4C*	9.16e‐31–2.22e‐18
Excitatory neurons set 3	*NDRG4, ADAM10, NFIX, RPLP1, GLCE, AK4, FBXW10, THSD7B*	2.22e‐30–8.82e‐06

*Note*: Genes with increased expression in female AD patients are depicted in red, while underexpressed genes are represented in blue.

Abbreviations: AD, Alzheimer's disease; DEGs, differentially expressed genes; FDR, false discovery rate; max, maximum; min, minimum; OPCs, oligodendrocyte precursor cells.

**TABLE 4:** Overview of the sex‐dimorphic DEGs with highest absolute log fold change (FDR  <  0.05), that is, representing the significant DEGs between AD patients and controls in both sexes, but with an opposite direction of the log fold change, studied at the level of individual cell types (left column).

Cell type	Gene symbol	Min and max FDR significance
Astrocytes	*CTNNA2, LAMA2, ZHX2, CTNNA3, GRID2, SORBS1, PALLD*	3.08e‐25–3.15e‐10
Immune cells	*CCL3, LINC02211, FBN2, BAG3*	3.02e‐34–7.62e‐09
Inhibitory neurons	*C5orf17, FOS, NR4A1, TBC1D3D, MTRNR2L8*	6.07e‐89–1.75e‐26
Oligodendrocytes	*AKAP6, SLIT2, PLCB1, OTUD7A, PCDH9, MOBP, RALYL*	2.53e‐11–8.11e‐09
Vasculature cells	*EGR1, SCN4A, C5orf17, SRPX2, PTGDR, PDGFD, KCNMA1, PRDM6*	2.25e‐21–3.03e‐02
OPCs	*C5orf17, TGFB2, KCNH1, LINGO1, RASGEF1B, SLC26A3*	6.24e‐13–4.45e‐03
Excitatory neurons set 1	*ZNF385D, PEX5L, TRPM3, RGS7, PDE4DIP, NRXN3*	1.23e‐19–3.64e‐17
Excitatory neurons set 2	*LDB2, ANKS1B, RTN1, CEMIP, HS3ST2, ADGRL3, ADGRB3*	5.50e‐29–3.09e‐15
Excitatory neurons set 3	*ZNF385D, GRID2, FGF14, SNTG1, HS3ST2, PHACTR1, PDE4DIP*	2.64e‐65–3.00e‐25

*Note*: Genes with increased expression in male AD patients (compared to male controls) are depicted in red, while genes with decreased expression in male AD patients (compared to male controls) are represented in blue. By definition of sex‐dimorphic genes, the opposite direction of change is observed when comparing female AD patients to female controls. For instance, if a sex‐dimorphic DEG is shown in red (increased in male AD vs. male controls), it exhibits decreased expression in female AD versus female controls.

Abbreviations: AD, Alzheimer's disease; DEGs, differentially expressed genes; FDR, false discovery rate; max, maximum; min, minimum; OPCs, oligodendrocyte precursor cells.

#### 3.1.1 Male‐specific DEGs

Among male‐specific DEGs, several genes stand out for their potential roles in AD pathology.

##### ERBB4

In astrocytes, the pronounced underexpression of *ERBB4* (ErB‐2 receptor tyrosine kinase 4; FDR = 3.19E‐293) is particularly noteworthy. This gene encodes a receptor tyrosine kinase that is essential for neurodevelopment and synaptic plasticity.^30^ Accumulating evidence has implicated *ERBB4* in AD, with altered expression potentially contributing to synaptic dysfunction and neuroinflammation.^31^ The gene is regulated by estrogen, and its promoter region contains a consensus estrogen response element half‐site that overlaps with activator protein‐1 binding sites.^32^ In response to estrogen stimulation, this regulatory element facilitates the binding of both the estrogen receptor and the *ERBB4* intracellular domain (4ICD), thereby modulating *ERBB4* expression. Given the estrogen‐responsive nature of *ERBB4*, the observed underexpression in male astrocytes may be attributed to lower levels of estrogen in males compared to females. Interestingly, a previous study on asymptomatic subjects with amyloid beta (Aβ) accumulation in the brain identified the *ERBB* signaling pathway as one of the key processes with sex‐differentiated expression, indicating that this pathway may play a role in sex‐specific differences in early asymptomatic stages of AD.^30^

##### GRM4

In excitatory neurons, the gene *GRM4* (glutamate metabotropic receptor 4) shows a male‐specific increased expression in AD (FDR = 1.02E‐51). Glutamate signaling is known to be influenced by sex hormones, and sex differences in the glutamate system have been reported in both human and animal model studies.^33^ Glutamate is essential for cognitive functions such as learning and memory, and AD is associated with both presynaptic and postsynaptic glutamatergic hypoactivity, which correlates strongly with cognitive decline.^34^ Moreover, a reciprocal relationship between glutamate and the Aβ protein in AD has been reported, in which Aβ can modulate glutamate levels at the synapse, and glutamate can alter the production of Aβ.^35^ In general, metabotropic glutamate receptors, such as the one encoded by *GRM4*, have been proposed as drug targets for neurodegenerative diseases to address pathological alterations in glutamatergic signaling.^36^ They may therefore also warrant further study in the context of addressing sex‐specific in the glutamate system in AD.

##### FTL

A significant male‐specific decreased expression in immune cells was observed for *FTL* (ferritin light chain; FDR = 2.67E‐84), encoding the light subunit of the ferritin protein. Previous studies on sex differences have demonstrated that ferritin expression serves as a reliable indicator of iron accumulation in tissues, and that ferritin levels tend to be higher in females compared to males across various age groups.^37^ *FTL* has been implicated in AD in the context of iron dysregulation, and increased iron accumulation in microglia in the cortex of AD patients has been found to be associated with increased *FTL* levels.^38^ Moreover, *FTL* mutations are associated with neurodegeneration, and *FTL* can interact with *PEN‐2* (presenilin enhancer 2) which serves as a regulatory component of the γ‐secretase complex involved in the cleavage of amyloid precursor protein (APP) to form Aβ.^39^ Overexpression of *FTL* has been found to increase levels of γ‐secretase components and promote Aβ production,^39^ indicating a potential causal link between altered *FTL* levels and Aβ accumulation as a hallmark of AD.

##### RPTOR

A male‐specific under‐expression was observed for *RPTOR* (regulatory associated protein of MTOR complex 1; FDR = 1.22E‐10) in astrocytes. *RPTOR* is a key regulator in the mechanistic target of rapamycin (mTOR) signaling pathway, which is known to display sex‐specific variations in models of AD pathology. For instance, a study found that female AD mice (APP/PS1) are protected from recall memory deficits and synaptic dysfunction associated with mTOR signaling until they reach 8 months of age, whereas male AD mice exhibit these deficits much earlier (at 2–4 months of age).^40^ After ovariectomy (surgical removal of ovaries, leading to an abrupt loss of estrogen), female AD mice showed deficits similar to young male AD mice, suggesting that estrogen has a protective role against AD pathology, specifically in delaying synaptic dysfunction and cognitive impairment mediated by Akt1–mTOR signaling.^41^ The mTOR pathway, including *RPTOR*, is critically involved in cellular metabolism, autophagy, and protein synthesis, which are key processes disrupted in AD. Hyperactivation of mTOR signaling has been observed early in AD progression, contributing to impaired autophagy, increased Aβ production, and tau pathology.^41^ Genetic reduction of mTOR signaling in an AD mouse model (Tg2576) reduced Aβ deposition and improved cognitive performance by enhancing autophagy, underscoring this pathway's role in disease pathogenesis.^41^

#### 3.1.2 Female‐specific DEGs

##### CCL2

Among female‐specific DEGs, the increased expression of *CCL2* (C‐C motif chemokine ligand 2; FDR = 3.19e‐30) in astrocytes is of particular interest. *CCL2*, encoding monocyte chemoattractant protein‐1, is a key regulator of neuroinflammation and has been implicated in AD pathogenesis.^42^ Moreover, *CCL2* was shown to be involved in sex‐specific neuroimmune responses in the hippocampus of male and female mice,^42^ matching with the sex‐dependent expression observed here. Interestingly, in immune cells, we observed that the closely related gene *CCL3* (C‐C motif chemokine ligand 3) displays a sex‐dimorphic alteration (with an increased expression in male AD, FDR = 1.61E‐53, and decreased expression in female AD, FDR = 2.53E‐15), indicating that similar neuroinflammatory pathways are altered across different cell types in AD, but in a cell type–specific manner.

##### INHBA

In inhibitory neurons, the gene *INHBA* (inhibin subunit beta A) stands out as a highly significant female‐specific DEG with increased expression in AD (FDR 2.23e‐64). *INHBA* encodes a subunit of activin A, a member of the transforming growth factor beta (TGF‐β) superfamily involved in neuroinflammation.^43^ Activin A is known to play a role in ovarian follicular development and hormone regulation, and overexpression of *INHBA* is known to decrease activin and estradiol secretion while increasing inhibin and progesterone secretion.^43^ Therefore, the joint involvement of *INHBA* in processes regulated by sex hormones and in neuroinflammatory and neuroprotective processes may explain the observed sex‐specific alterations in AD.

##### FGF1

The fibroblast growth factor *FGF1* displayed a female‐specific under‐expression in AD (FDR = 1.20E‐31) in vascular cells. Sex hormone regulation of *FGF1* expression has previously been demonstrated in several tissue types,^44^ though not specifically in the context of AD and brain disorders. *FGF1* exerts neuroprotective effects against glutamate toxicity by inactivation of the glycogen synthase kinase‐3β (GSK3β) pathway through the PI3K–Akt cascade.^45^ Because the overactivity of GSK3β in AD is considered a key contributor to AD pathophysiology,^45^ *FGF1* may warrant further investigation as a therapeutic target for GSK3β‐mediated neuroprotection in AD.

##### LEPR

The leptin receptor gene *LEPR* showed a female‐specific decreased expression in AD (FDR = 6.65E‐31) in immune cells. Sex hormone regulation of leptin signaling has been well established, with leptin levels and sensitivity showing sexual dimorphism.^46^ In the context of AD, leptin signaling has been implicated in neuroprotection.^46^ Specifically, leptin has been shown to attenuate tau hyperphosphorylation in neuronal cells and to reduce Aβ protein levels.^47^ Moreover, evidence of leptin resistance has been found in AD brains, with increased leptin levels but decreased leptin receptor expression.^47^ This dysregulation of leptin signaling may contribute to AD pathological changes. Given the role of leptin in neuroprotection and its sex‐specific regulation, *LEPR* may warrant further investigation as a potential target for sex‐specific therapeutic strategies in AD.

#### 3.1.3 Sex‐dimorphic DEGs

The differential expression analysis highlighted several genes that are significantly altered in both sexes but with opposite directions of the change.

##### EGR1

In vascular cells, *EGR1* (early growth response 1; FDR 1.75E‐19) is identified as a significant sex‐dimorphic DEG. This immediate early gene encodes a zinc‐finger transcription factor involved in immune response and tissue injury processes and is also known as a regulator of synaptic plasticity and neuronal activity.^48^ The sex‐dependent profile matches with the fact that *EGR1* expression is regulated by 17β‐estradiol, a sex hormone that contributes to sexual dimorphism.^49^ Women, who tend to have higher circulating levels of 17β‐estradiol, may therefore have different patterns of *EGR1* expression compared to men. Interestingly, we have previously already observed significant sex‐dimorphic changes in *EGR1* orthologs in two mouse models of early AD‐like pathology: the THY‐Tau22 model of tauopathy^50^ and the Tg2576 model of Aβ pathology. This suggests that *EGR1* might also play an important role in the early stages of AD pathology. In addition, *EGR1* has been linked to human AD pathology due its known role in the stimulation of acetylcholinesterase (AChE) expression.^50^ Increased AChE activity in AD can accelerate the degradation of acetylcholine, an essential neurotransmitter for cognitive function. Thus, sex‐dimorphic expression of *EGR1* in AD may contribute to differences in cholinergic function between male and female patients. Furthermore, recent studies using the 3xTg‐AD mouse model have shown that suppressing the expression of the mouse *EGR1* ortholog in the hippocampus reduces tau phosphorylation, decreases Aβ pathology, and improves cognition.^50^ Because human EGR1 stimulates presenilin‐2 in neuronal cells, its inhibition may also reduce amyloidogenic processing of APP. While these findings point to *EGR1* inhibition as a potential strategy to address cholinergic deficits in AD, *EGR1* also has significant physiological roles, for example, in memory formation, as we noted in our previous Tg2756 model study.^50^ Thus, further research is warranted to evaluate whether partial *EGR1* inhibition could be a viable pharmacological strategy in AD.

##### FOS

In inhibitory neurons, *FOS* (proto‐oncogene C‐fos) displayed a significant sex‐dimorphic pattern, with increased expression in male AD (FDR = 7.3E‐120) and decreased expression in female AD (FDR = 6.07E‐89). *FOS* is regulated by estrogen and is involved in the sexual differentiation of the brain, explaining its sex‐dimorphic expression patterns. In the context of AD, *FOS* has previously already been reported to be overexpressed in hippocampal neurons in AD^51^ and as an immediate early gene; it is thought to be required for the initiation of apoptosis in AD.^51^ Furthermore, hippocampal *FOS* expression is essential for memory formation. Interestingly, aberrant *FOS* expression was observed already at the pre‐plaque stage in cortical regions of a J20 mouse model of AD, suggesting that it reflects early stages of pathology.^52^

##### NR4A1

Another sex‐dimorphic DEG in inhibitory neurons is the nuclear hormone receptor *NR4A1* (increased in male AD with FDR = 2.56E‐50, and decreased in female AD with FDR = 1.75E‐20). This gene is regulated by sex hormones and plays a role in the hypothalamic–pituitary–gonadal axis, indicating its involvement in sex‐dependent physiological functions.^53^ Like other NR4A orphan nuclear receptors, *NR4A1* is a mediator of cyclic adenosine monophosphate response element binding protein (CREB)‐dependent neuroprotection and has been proposed as a potential therapeutic target for AD, as its ortholog's pharmacological activation in mice was shown to rescue age‐associated memory decline.^53^

##### FKBP5

In oligodendrocytes, a sex‐dimorphic alteration was observed for *FKBP5* (FK506 binding protein 5), with increased levels in male AD (FDR = 1.17E‐295) and decreased expression in female patients (FDR = 1.06E‐294). *FKBP5* encodes a co‐chaperone in the glucocorticoid receptor complex and is regulated by glucocorticoids and androgens,^54^ in line with its sex‐dependent expression pattern. When genotyping *FKBP5* in an AD cohort, a synergistic effect of its alleles and the *APOE* genotype on anxiety scores and a sex‐dependent effect of *FKBP5* promoter methylation on neurophysiological brain activity has been observed.^55^ Furthermore, the chaperone complex formed between *FKBP5* and *HSP90* has been shown to prevent degradation of the microtubule‐associated protein tau,^56^ whose oligomerization is a key hallmark of AD.

**FIGURE 1:**
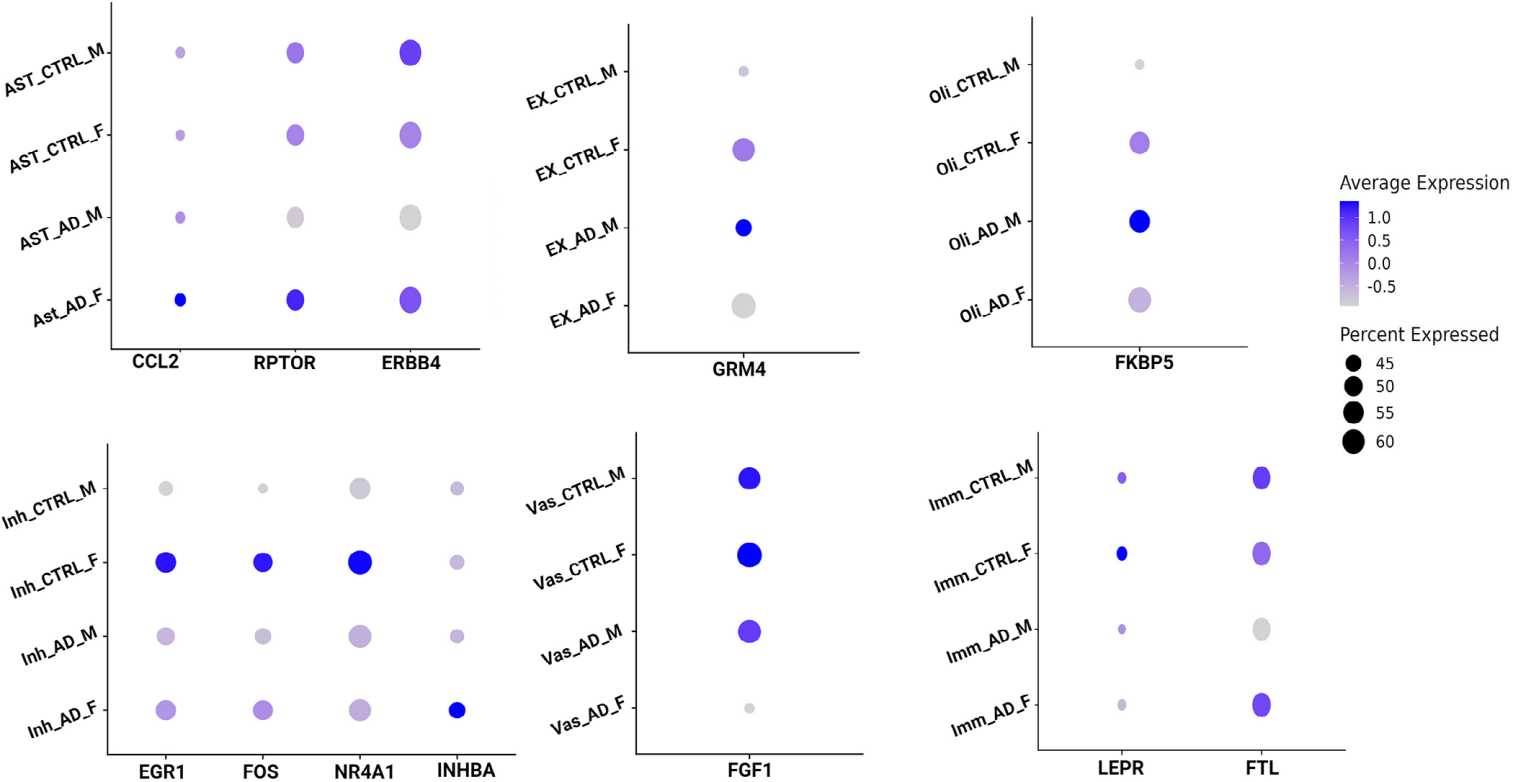
Dot plots showing the expression of key genes across specific cell types in AD patients and controls. The size of each dot represents the percentage of cells expressing the gene, while the color intensity indicates the average expression levels between females and males with AD across multiple cell types. The *y* axis shows the cell types, while the *x* axis displays the gene symbols. AD, Alzheimer's disease; ST, astrocytes; CTRL, healthy control; EX, excitatory neurons; F, female; Imm, immune cells; Inh, inhibitory neurons; M, male; Oli, oligodendrocytes; Vas, vascular cells.

Figure 1 illustrates the expression levels of the key sex‐dependent DEGs discussed above for all combinations of conditions and sexes, highlighting the diverse patterns of sex‐dependent alterations observed in the data. In Figure S1 in supporting information we additionally visualize the expression of these key DEGs stratified by condition, sex, and Braak stage.

Overall, these findings of statistically significant sex‐dependent alterations in multiple genes with established functional roles in AD‐related processes highlight the importance of considering sex as a biological variable in AD research. For further investigation into the functional consequences of these sex‐specific and sex‐dimorphic expression patterns, pathway enrichment analyses and network analyses were conducted (see following sections).

#### 3.1.4 Sex‐dependent changes in mitochondrial genes

Mitochondrial dysfunction is a well‐established feature of AD pathology, with impaired energy metabolism and increased oxidative stress contributing to neuronal death and cognitive decline. Given the central role of mitochondria in cellular energy production and the high energy demands of the brain, genes encoding mitochondrial proteins may be particularly relevant for understanding sex differences in AD progression and manifestation.

Mitochondrial genes fall into two categories: those encoded by mitochondrial DNA (mtDNA) and nuclear‐encoded mitochondrial genes. mtDNA‐encoded genes are of particular interest because mitochondria are maternally inherited, potentially contributing to sex‐specific effects. In addition, mtDNA is more susceptible to oxidative damage than nuclear DNA, which may be relevant to AD pathology. Nuclear‐encoded mitochondrial genes, while following Mendelian inheritance, may still exhibit sex‐specific expression patterns due to hormonal influences or other sex‐dependent regulatory mechanisms. While our analysis below focuses on mtDNA‐encoded genes due to their maternal inheritance pattern, we provide the complete statistics for nuclear‐encoded mitochondrial genes as Table S3 in supporting information. Several mtDNA‐encoded genes showed significant sex‐dependent differential expression in multiple cell types in AD brains (see Table S4 in supporting information for detailed statistics).

In astrocytes, we observed a male‐specific significant under‐expression of *MT‐CO1* (mitochondrially encoded cytochrome C oxidase I; FDR = 8.198E‐116). *MT‐CO1* is a key component of the mitochondrial electron transport chain, particularly complex IV. Its male‐specific change suggests potential sex‐specific impairments in cellular respiration and energy production in astrocytes from male AD patients.

We also identified many sex‐dimorphic mtDNA‐encoded genes in astrocytes, including *MT‐ND3, MT‐ND1*, *MT‐ND2*, *MT‐CYB, MT‐CO3*, and *MT‐CO2*. These genes showed significant differential expression in both sexes, but with more pronounced changes in males (FDR = 3.192E‐293 for all three genes in males). *MT‐ND3*, *MT‐ND2*, and *MT‐ND1* are components of complex I of the electron transport chain, *MT‐CYB* is a component of complex III, while *MT‐CO3* and *MT‐CO2* are part of complex IV. The dimorphic expression of these genes indicates that while mitochondrial function is altered in astrocytes of both sexes in AD, the degree and possibly the consequences of these alterations may differ between females and males.

In excitatory neurons, we observed sexually dimorphic expression of several mtDNA‐encoded genes, including *MT‐ND5, MT‐ND2, MT‐ND1, MT‐ND4, MT‐ND3, MT‐ND4L, MT‐ATP8*, and *MT‐CYB*. All of these genes showed highly significant differential expression in both sexes but with opposite directions of change, with a decreased expression in males as opposed to increased expression in females, except for *MT‐ND5* with changes in the opposite directions. This striking pattern points to fundamentally different mitochondrial responses or adaptations in excitatory neurons of male versus female AD patients, mainly affecting complex I genes. Given the critical role of these genes in the electron transport chain, these sex‐specific changes could lead to divergent energy metabolism profiles in neurons, potentially contributing to sex differences in AD progression or symptomatology.

In oligodendrocytes, we again detected sexually dimorphic expression of numerous mtDNA‐encoded genes, including mostly complex I genes (*MT‐ND1, MT‐ND2, MT‐ND3*, and *MT‐ND4*, all of which have increased expression in males), but also a member of complex IV (*MT‐CO3*, increased in males) and complex V (*MT‐ATP6*, decreased in males). As with excitatory neurons, these genes showed highly significant differential expression in both sexes, but with more pronounced changes in males. The low FDR values in both sexes indicate robust and widespread changes to mitochondrial gene expression in oligodendrocytes, highlighting the importance of mitochondrial dysfunction in AD pathology across sexes.

In vascular cells, we identified only a male‐specific significant underexpression of *MT‐ND4L* (FDR = 4.47E‐05). While this finding will require further confirmation, it indicates potential sex‐specific impairments in mitochondrial function in the vasculature of male AD patients, which may contribute to sex differences in vascular contributions to AD pathology.

Collectively, these findings highlight widespread sex‐dependent alterations in mtDNA‐encoded mitochondrial gene expression across multiple cell types in AD. The observed patterns suggest that while mitochondrial dysfunction is a common feature of AD, it may manifest differently in males and females.

### 3.2 Sex‐dependent gene set enrichment analysis

To gain a deeper understanding of the biological processes and pathways affected by sex‐specific gene expression changes in AD, we performed gene set enrichment analysis (GSEA) on the identified sex‐dependent DEGs. GSEA allows us to go beyond individual gene changes and identify coordinated changes in functional gene sets and pathways that may be relevant for AD pathogenesis. By performing separate enrichment analyses for male‐specific, female‐specific, and sex‐dimorphic gene sets in different cell types, we aimed to detect cell type–specific sex‐dependent biological processes in AD. The following sections detail our main findings for key cell types involved in AD pathology with large cell counts in the data, highlighting the most significantly enriched pathways and their potential implications for sex‐specific disease mechanisms (complete pathway ranking tables are provided in Table S5 in supporting information).

#### 3.2.1 Astrocytes: male‐specific changes

**FIGURE 2:**
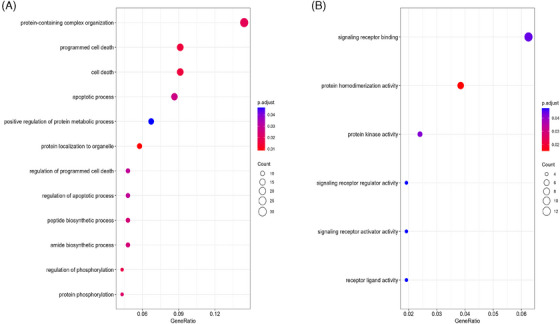
Enriched GO terms in male astrocytes, representing the significant terms in males (*P* adjusted <  0.05) which do not approach significance in females (*p*  >  0.1). A, Enriched GO biological processes in males. B, Enriched GO molecular functions in males. GO, Gene Ontology.

GSEA of male astrocytes in AD revealed significant alterations in several biological processes and molecular functions (Figure 2A,B). Most notably, we confirmed a pronounced enrichment of cell death–related processes, such as programmed cell death (GO:0012501), cell death (GO:0008219), and apoptotic process (GO:0006915). These pathways showed the highest gene ratio and statistical significance (adjusted *P* value < 0.05) among the enriched terms, suggesting a potential increased vulnerability of male astrocytes to AD‐related cellular stress.

The analysis also highlighted enriched changes in protein metabolism and complex organization pathways, such as protein‐containing complex organization (GO:0043933) and positive regulation of protein metabolic process (GO:0032270). This indicates a sex‐specific dysregulation of protein homeostasis, which aligns with previous studies emphasizing the importance of protein misfolding and aggregation in AD pathogenesis, particularly in astrocytes that play an essential role in maintaining brain homeostasis.

In terms of molecular functions (Figure 2B), the enrichment analysis revealed significant alterations in signaling receptor binding (GO:0005102), protein kinase activity (GO:0004672), and signaling receptor activator activity (GO:0038023). These results suggest potential changes in astrocyte–neuron communication and intracellular signaling cascades, which are central aspects of AD pathology.^57^ Corresponding female‐specific pathway alterations in astrocytes are presented in the following section and in Figure S2 in supporting information.

#### 3.2.2 Astrocytes: female‐specific changes

The GSEA of female astrocytes in AD revealed distinct patterns of pathway alterations compared to males. Notably, there was significant enrichment in the regulation of the canonical Wnt signaling pathway (GO:0060828, adjusted *P* value = 0.02). This pathway, important for neuronal survival and synaptic plasticity, involves key female‐specific DEGs such as *BICC1*, *SOX9, USP47, RBPJ, MCC*, and *KPNA1*. This finding aligns with previous studies indicating that Wnt signaling may have neuroprotective effects in AD and could contribute to observed sex differences in disease progression.

The analysis also highlighted enrichment in cell cycle–related processes, particularly negative regulation of G1/S phase transition (GO:1902807, adjusted *P* value = 0.02). This pathway covers genes *CCL2*, *CRLF3*, and *RB1*, suggesting potential alterations in cell proliferation that may be specific to female astrocytes in AD.

Importantly, the enrichment analysis revealed significant changes in locomotion‐related pathways (GO:0040011, GO:0040012, GO:2000145, all with adjusted *P* value = 0.03). These pathways involve several significant female‐specific DEGs including *CCL2, PTPRJ, RGN, ADAMTS9, LRP1, SOX9, CLASP2, CCDC125, NF1, RIPOR2, MCC, ITGA5, SDCBP*, and *ANGPT2*. This indicates that female astrocytes in AD may undergo distinct changes in their ability to move and interact with the surrounding environment. These sex‐specific alterations could affect how astrocytes migrate through brain tissue and engage with the extracellular matrix, potentially influencing their role in disease progression.

Other significantly enriched pathways with female‐specific alterations include regulation of cell migration (GO:0030334, adjusted *P* value = 0.04) and intracellular signal transduction (GO:0035556, adjusted *P* value = 0.04), further emphasizing the complex changes in cellular behavior and signaling in female astrocytes in AD.

An overview of the most significant female‐specific enriched GO biological processes in astrocytes, analogous to the male‐specific results shown in Figure 2, is presented in Figure S2, with detailed results provided in Table S5.

#### 3.2.3 Excitatory neurons: male‐specific changes

The analysis of male‐specific changes in excitatory neurons highlighted significant alterations in several key cellular processes, including apoptosis regulation. Notably, there was enrichment in the GO term negative regulation of apoptotic process (GO:0043066, adjusted *P* value = 0.02), involving DEGs such as *IFIT3, ESR1, SLC25A4, TNFAIP8*, and *NES*. This may indicate a potential compensatory mechanism in male neurons to counteract AD‐related stress and cell death. The involvement of the mitochondrial ADP:ATP antiporter *SLC25A4* in this pathway is particularly interesting, as its altered expression could be linked to mitochondrial function, energy metabolism, and potentially glutamate regulation, all of which are relevant to AD pathology.

Furthermore, there was enrichment in pathways related to the regulation of kinase activity (GO:0043549, adjusted *P* value = 0.003; GO:0045859, adjusted *P* value = 0.003; GO:0033674, adjusted *P* value = 0.004; GO:0045860, adjusted *P* value = 0.004). These pathways involve DEGs such as *MAP2K3, SYAP1, TENM1*, and *CAMKK2*, suggesting alterations in signaling cascades that control neuronal function and survival. More specifically, there was also a significant enrichment in the positive regulation of MAPK cascade (GO:0043410, adjusted *P* value = 0.02), involving *GRM4, MAP2K3, TENM1*, and *MADD*. This aligns with previous findings linking MAPK signaling to AD pathology, particularly in mediating the effects of Aβ on synaptic function and plasticity.

The analysis also highlighted enrichment in cell–cell signaling pathways (GO:0007267, adjusted *P* value = 0.006) and more specifically in synaptic signaling pathways (GO:0099537, GO:0007268, GO:0099536, all with adjusted *P* value = 0.02), involving the male‐specific DEGs *GRM4, TLE2, DLGAP4, ALOX5, SYAP1*, and *TM7SF3*. This highlights changes in neuronal communication and synaptic function in male AD neurons.

#### 3.2.4 Excitatory neurons: female‐specific changes

Female excitatory neurons in AD exhibit a unique profile of pathway alterations. Given the established association between Aβ accumulation and increased signal transducer and activator of transcription 3 (STAT3) phosphorylation in AD,^58^ a particularly noteworthy finding is the enrichment of genes involved in the positive regulation of tyrosine phosphorylation of STAT protein (GO:0042531, adjusted *P* value = 0.05). This pathway covers multiple female‐specific DEGs, such as *FLT3, HPX*, and *HCLS1*, indicating that alterations in STAT signaling in AD neurons display a broad female‐specific pattern.

The analysis also highlighted a significant enrichment in the regulation of synaptic plasticity (GO:0048167, adjusted *P* value = 0.01), involving *GRIN2A, SLC38A1, SLC24A1*, and *KCNJ10*. This suggests potential alterations in synaptic function and plasticity specific to female AD neurons.

Interestingly, there was significant enrichment in ossification‐related pathways (GO:0001503, adjusted *P* value = 0.02), involving DEGs such as *DDR2, ENPP1, TNFAIP6, PENK, DHX36, FAM20C, CCDC134, TUFT1, WNT5A, SHH*, and *SEMA4D*. While seemingly unrelated to AD, this enrichment may reflect broader dysregulation of calcium homeostasis and related signaling pathways in AD,^59^ as calcium plays a central role in both bone formation and neuronal function. Of particular interest is the role of *DHX36* in translation regulation and synaptic plasticity, as its knockdown was shown to affect dendritic morphogenesis and brain‐derived neurotrophic factor (BDNF)‐induced dendritic changes.^60^

The analysis also revealed enrichment in chemotaxis and taxis pathways (GO:0006935 and GO:0042330, both with adjusted *P* value = 0.01), indicating potential changes in cellular migration and response to external stimuli in female AD neurons. This finding was supported by several female‐specific DEGs, including *NRG3, FER, SEMA3A, PIK3C2G, CORO1A, TNFAIP6, PLAUR, WNT5A, OXSR1, PTPN2, RHOG, IL17RA*, and *SEMA4D*.

Additionally, there was an overrepresentation of female‐specific DEGs in the regulation of cell division (GO:0051302, adjusted *P* value = 0.001) and more specifically, positive regulation of cell division (GO:0051781, adjusted *P* value = 0.003), involving DEGs such as *PKN2, KIF14, TLE6*, and *SHH*. This more unexpected finding might indicate alterations in cell cycle–related processes in female AD neurons.

Overall, the comprehensive nature of these changes, spanning from signaling pathways to synaptic plasticity and cell division, underscores the complex impact of AD on female excitatory neurons.

#### 3.2.5 Vascular cells: male‐specific changes

Male‐specific alterations in vascular cells associated with AD reveal a complex interplay of cellular processes. The most significant changes involve the transmembrane receptor protein serine/threonine kinase signaling pathway (GO:0007178) and its regulation (GO:0090092, both adjusted *P* value = 0.02), which are part of the broader TGF‐β receptor superfamily signaling pathway (GO:0141091). These pathways involve key DEGs such as *RGMA, SINHCAF, ZBTB7A, FOXD1*, and *TGFB1*.

Further significant male‐specific changes were observed in pathways regulating cellular movement and response to stimuli. These include the negative regulation of locomotion (GO:0040013) and regulation of chemotaxis (GO:0050920, both adjusted *P* value = 0.03), involving DEGs such as *RNF41, SEMA3F, SINHCAF, TGFB1, FGF2, NOVA2*, and *GAS6*. The involvement of *FGF2* is of particular interest given its role in microglial activation, and its previously reported potential to restore spatial learning and neurogenesis in AD models.^61^ Closely related are changes in the pathways regulation of cell migration (GO:0030334) and regulation of cell motility (GO:2000145, both adjusted *P* value = 0.02), which additionally involve the DEGs *SCARB1, SYDE1*, and *CASS4*. Moreover, the broader pathway negative regulation of response to stimulus (GO:0048585, adjusted *P* value = 0.007) was significantly enriched, including many of the above DEGs plus others, such as *FZD9, TNFAIP8L1, UFD1, INPP5D*, and *GRK2*. This suggests a wide‐ranging impact on how vascular cells in male AD patients respond to their environment.

The cell surface receptor signaling pathway (GO:0007166, adjusted *P* value = 0.01) emerged as another significantly altered process, involving an extensive set of DEGs including, in addition to those mentioned above, the genes *ADGRE5, IL17RA, LRP8, DTX3, STAT5B, SH2B3*, and *VPS4B*. These findings indicate that male‐specific vascular changes in AD extend beyond traditional vascular functions, potentially influencing cell migration, chemotaxis, and responses to various stimuli.

Overall, the enrichment of transmembrane receptor and TGF‐β signaling pathways points to alterations in important cellular communication processes, which could affect vascular function, neuroinflammation, and neuronal health in male AD patients. Moreover, the changes in locomotion and chemotaxis regulation suggest impacts on cellular movement and environmental responsiveness, potentially influencing vascular remodeling and inflammatory responses in the AD brain.

#### 3.2.6 Vascular cells: female‐specific changes

Vascular cells in female AD patients displayed a distinctive profile of enriched pathways, revealing significant alterations across several interconnected cellular processes. Strikingly, the most significant changes were observed in pathways governing signal transduction and cell communication. Positive regulation of signal transduction (GO:0009967), cell communication (GO:0010647), and signaling (GO:0023056) all showed high enrichment (adjusted *p* value = 0.003), involving DEGs such *FGF1, CNTN6, TCIM, LAMB1*, and *FERMT1*. The general regulation of these processes (GO:0010646, GO:0023051) was also significantly enriched (adjusted *P* value = 0.01).

Pronounced alterations were also observed in pathways related to kinase regulation, including regulation of kinase activity (GO:0043549), protein kinase activity (GO:0045859), and transferase activity (GO:0051338, all with adjusted *P* value = 0.02). These pathways include DEGs with highly significant changes, such as *PPM1E, FGF1*, and *TCIM*. Closely related to kinase regulation, protein phosphorylation (GO:0006468) and broader protein modification processes (GO:0036211, GO:0043412) also showed significant alterations (adjusted *P* value = 0.03), involving additional DEGs such as *GRK3*.

Furthermore, pathways related to positive regulation of metabolic processes (GO:0009893, GO:0010604, GO:0031325) and regulation of protein metabolism (GO:0051246) displayed strong changes (adjusted *P* value = 0.03), primarily involving the DEGs *FGF1, TCIM*, and *FERMT1*. The recurrent involvement of *FGF1* across multiple pathways underscores its potential significance in female‐specific AD pathology in vascular cells. Its known neuroprotective roles, possibly mediated through regulation of GSK3β, may be particularly relevant in the context of vascular contributions to AD pathology.

The enrichment of protein phosphorylation and kinase regulation pathways, especially involving *PPM1E*, suggests alterations in signaling cascades that could affect synaptic plasticity and dendritic spine morphogenesis. PPM1E, a brain‐specific phosphatase previously linked to AD, points to a complex interplay between vascular cells and neuronal function that may be uniquely altered in female AD patients.^62^ Taken together, the significant changes in signal transduction and cell communication pathways indicate broader alterations in how vascular cells respond to and transmit signals in the AD brain.

### 3.3 Cell–cell communication analysis

Using the integrative computational approach for analyzing intercellular communication (see Methods section), we identified multiple significant patterns of distinct cell–cell signaling in male and female AD, showing that cell–cell communication events in AD display pronounced and significant sex dependencies.

#### 3.3.1 Male‐specific communication patterns

In male AD, significant changes in cell–cell communication events were mainly observed in astrocytes and excitatory neurons and mostly associated with apoptotic processes. This finding aligns with our pathway enrichment and gene‐level differential analysis results, highlighting the importance of cell death pathway alterations in male AD.

##### Astrocytes

**FIGURE 3:**
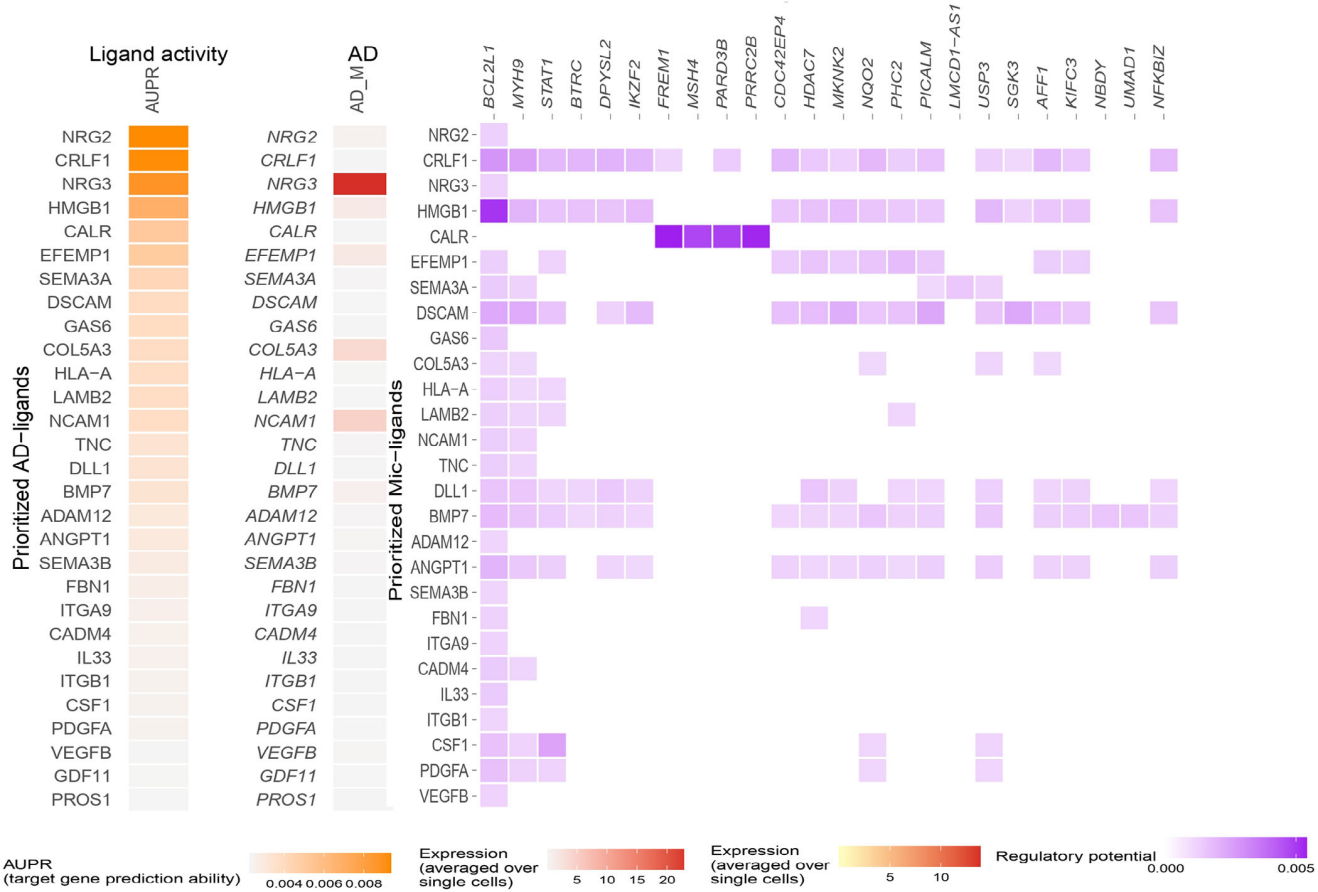
Cell–cell communication analysis results for male astrocytes, highlighting the top 30 ligands and their downstream targets. Associated enriched gene ontology terms between the different cell type clusters are shown in Table S6 in supporting information. The cell–cell communication analysis results for female astrocytes are presented in Figure S3 in supporting information.

In male astrocytes, cell–cell communication analysis revealed altered signaling events significantly affecting apoptotic processes. These include the dendritic cell apoptotic process (GO:0097048, adjusted *P* value: 0.03) and apoptotic process involved in morphogenesis (GO:0060561, adjusted *P* value: 0.003). The affected processes involve multiple ligand–receptor interactions, highlighting the complexity of cell death regulation in astrocytes. Aberrant apoptosis contributes to neuronal loss in AD,^63^ and sex hormones have been reported to differentially regulate apoptotic processes. These findings are consistent with the male‐specific underexpression of the apoptosis‐associated genes *RPTOR* (FDR = 1.22E‐10) and *ERBB4* (FDR = 3.19E‐293) in astrocytes. Moreover, immune system and cell movement–related processes such as lymph vessel morphogenesis (GO:0036303, adjusted *P* value: 0.03) and dendritic cell migration (GO:0036336, adjusted *P* value: 0.03) were significantly affected. Top ranked ligand activities and their downstream targets associated with the mentioned biological processes in astrocytes are illustrated in Figure 3, and detailed statistics for all GO terms across all cell types are provided in Table S7 in supporting information.

##### Excitatory neurons

Male excitatory neurons showed enrichment in pathways related to the negative regulation of programmed cell death (GO:0043069, adjusted *P* value: 0.008). Similar to the alterations in cell death pathways observed in astrocytes, sex hormones can differentially regulate the corresponding pathways in excitatory neurons as well.^63^ Three genes involved in the relevant ligand–receptor interactions, *IGF1R, TYRO3* and *ERBB4*, were also identified as significantly differentially expressed in male excitatory neurons, with sex‐dimorphic changes for *IGF1R* and *TYRO3I*, and a sex‐shared change for *ERBB4*. Furthermore, changes in cell–cell communication affected processes related to the organization and shaping of cellular structures, such as regulation of anatomical structure morphogenesis (GO:0022603, adjusted *P* value: 2.5E‐11) and postsynaptic membrane assembly (GO:0097104, adjusted *P* value: 5.4E‐09).

##### Inhibitory neurons

In male inhibitory neurons, the significant pathways highlight in particular changes in synaptic membrane organization and Ras signaling. They include the pathway postsynaptic membrane assembly (GO:0097104, adjusted *P* value: 3.81E‐09), which has been implicated in synaptic dysfunction as a key feature of AD. The observation of male‐specific synaptic changes is confirmed by the second significant pathway, presynaptic membrane organization (GO:0097090, adjusted *P* value: 3.18E‐07). Alterations in presynaptic genes have indeed been proposed to contribute to synaptic pathology in AD, as both Aβ and tau are known to target presynaptic and postsynaptic proteins.^64^

A third significant pathway, positive regulation of Ras protein signaling (GO:0046579, adjusted *P* value: 0.001), has also been implicated in AD pathology, particularly in relation to neuroinflammation and neuronal survival. Specifically, oligomeric Aβ has been reported to hyperactivate Ras, which prevents N‐methyl‐D‐aspartate receptor–dependent long‐term potentiation and ultimately results in impaired cognition.

##### Oligodendrocytes

In male oligodendrocytes, the pathway most significantly affected by cell–cell communication changes is positive regulation of cellular component organization (GO:0051130, adjusted *P* value: 1.95E‐05). It involves multiple genes that have been directly implicated in AD pathology, such as *APP* (involved in Aβ production), *IGF1R* (involved in cell survival and metabolism), and *MTOR* (involved in protein synthesis and autophagy regulation). All these genes display significant sex‐dependent patterns in oligodendrocytes, with sex‐dimorphic changes for *APP* (FDR = 1.17E‐295) and *IGF1R* (FDR = 1.17E‐295) and a male‐specific alteration for *MTOR* (FDR* = *1.41E‐79). Moreover, these three genes are all known to be regulated by sex hormones,^65^ suggesting a potential mechanism for the observed sex‐specific effects in AD.

##### Vascular cells

Cell–cell communication analysis in male vascular cells revealed altered signaling events affecting the myeloid cell apoptotic process (GO:0033028), involving the transcription factor *STAT5B* and the ligand *GAS6*, which are both male‐specific DEGs. The alteration of this pathway in vascular cells may indicate that vascular contributions to AD pathology in males involve modulation of immune cell survival. This matches with the fact that myeloid cell apoptosis has already been implicated in AD pathogenesis, and more generally, sex differences in immune responses have also been described in AD.^66^

Furthermore, the G protein‐coupled acetylcholine receptor signaling pathway (GO:0007213) was identified as impacted in vascular cells, involving the male‐specific DEG *GRK2*, which encodes a central kinase in this pathway. This finding is in line with the well‐established association between cholinergic deficits and AD and previously reported sex differences in cholinergic system function in AD.^67^

Finally, the pathway maintenance of synapse structure (GO:0099558) is also significantly affected by cell–cell communication changes, matching with the significant findings for synaptic pathways in other cell types. Indeed, synaptic dysfunction is one of the main hallmarks of AD, and sex differences in synaptic plasticity have been observed in the disease.^68^

#### 3.3.2 Female‐specific communication patterns

##### Astrocytes

Cell–cell communication analysis for female astrocytes revealed significant alterations in intercellular signaling impacting Wnt signaling (GO:0090263, positive regulation of the canonical Wnt signaling pathway, adjusted *P* value* = 0.014*). Given that altered Wnt signaling has been associated with increased Aβ production and tau phosphorylation in AD,^69^ this finding may point to a relevant driving mechanism for AD pathology and progression rather than a downstream effect of the disease. Moreover, estrogen is known to modulate Wnt signaling,^70^ suggesting a potential mechanism for sex‐specific effects in AD. The results also complement the female‐specific increased expression observed for one of the top‐ranked DEGs in astrocytes, *CCL2* (FDR = 3.24E‐101, see above), as *CCL2* can interact with Wnt signaling in neuroinflammatory contexts.

Furthermore, the analysis highlighted altered intercellular signaling impacting the C‐Jun N‐terminal kinase (JNK) cascade (GO:0007254) in female astrocytes, aligning with previous studies implicating JNK signaling in Aβ‐induced neurotoxicity and tau phosphorylation in AD^71^ and the differential regulation of this pathway by sex hormones.^71^ For illustration purposes, Figure S3 in supporting information highlights the most significantly altered ligand activities and their associated downstream targets in astrocytes, which are linked to the biological processes discussed above. The detailed results are included in Table S7.

##### Excitatory neurons

In female excitatory neurons, the pathway regulation of calcium ion transport into cytosol (GO:0010522, adjusted *P* value = 0.02) was significantly impacted by altered cell–cell communication. Dysregulation of calcium homeostasis is a well‐established characteristic of AD, as shown by numerous studies linking altered calcium homeostasis to synaptic dysfunction and neuronal death in AD.^72^ Moreover, sex hormones, particularly estrogen, modulate calcium signaling in neurons, potentially contributing to sex differences in AD. This matches with the fact that PIK3 genes such as *PIK3C2G*, identified as one of the top‐ranked female‐specific DEGs in excitatory neurons (FDR = 1.9E‐58), can modulate calcium channel trafficking and calcium signaling.

##### Inhibitory neurons

Multiple pathways with female‐specific significance in inhibitory neurons are associated with both AD and sex differences. The most significant one, peptidyl‐tyrosine autophosphorylation (GO:0038083, adjusted *P* value = 8.4E‐06), involves the receptors *EGFR* and *IGF1R* that have both been implicated in AD pathology. In particular, suppression of *IGF1R* signaling in neurons has been shown to protect against neuroinflammation, anxiety, and memory impairments caused by Aβ oligomers. *EGFR* inhibitors were reported to attenuate Aβ pathology and improve cognitive function in AD mouse models. Moreover, both *IGF1R* and *EGFR* signaling show sex‐specific differences and are regulated by sex hormones.^73^

The second most significant pathway, trans‐synaptic signaling, modulating synaptic transmission (GO:0099550, adjusted *P* value = 0.0002) is essential for synaptic function. Synaptic dysfunction is a hallmark of AD, and alterations in trans‐synaptic signaling can impair communication between neurons, potentially contributing to the cognitive decline in AD. In addition, synaptic plasticity and neurotransmission show sex‐specific differences that may be relevant to AD progression.^31^

Interestingly, the significant pathways also included steroid hormone processes, such as response to testosterone (GO:0033574, adjusted *P* value = 0.03) and the more generic process response to steroid hormone (GO:0048545, adjusted *P* value = 0.04). Steroid hormones, particularly estrogen and testosterone, have been implicated in AD risk and progression in several studies, most of which highlighted protective effects.^32^, ^44^, ^46^ The modulation of these inherently sex‐specific pathways in female inhibitory neurons suggests that these cells may be particularly responsive to hormonal changes.

##### Oligodendrocytes

Analysis of female‐specific pathways impacted in oligodendrocytes highlighted two key processes with strong evidence for both sex differences and AD associations. The first pathway, sequestering of calcium ion (GO:0051208, adjusted *P* value = 0.0008) involves the female‐specific DEGs *CALR*, *CALM3*, and *HSP90B1*; the sex‐shared DEGs *CALM1* and *RYR2*; and the sex‐dimorphic DEG *ITPR1*. As already pointed out above, calcium dysregulation is a well‐established feature of AD pathology,^60^ and estrogen is known to modulate calcium signaling.

The second prominent pathway is cellular response to lipid (GO:0071396, adjusted *P* value = 0.005), which includes genes such as the female‐specific DEG *CALR*, the male‐specific DEG *ADAM9*, and the sex‐dimorphic gene *ITGA2*. Changes in lipid metabolism have been implicated in AD pathogenesis, with significantly altered lipid profiles observed in multiple tissues and body fluids. In addition, sex hormones are known to influence lipid metabolism, and may therefore contribute to these particular sex differences in AD. Given the essential role of lipids in myelin composition, sex‐specific alterations in lipid response pathways may have significant implications for oligodendrocyte function and myelination in the context of AD.

##### Vascular cells

In female vascular cells, altered signaling events showed a significant impact on the notch signaling pathway (GO:0007219, adjusted *P* value = 2.2E‐07). Notch signaling is involved in processes such as neurogenesis, synaptic plasticity, and neuronal survival, all of which are modulated in AD.^49^ Moreover, notch signaling interacts with estrogen signaling, suggesting a potential mechanism for sex‐specific patterns in this pathway.^74^ This pathway finding also matches with the female‐specific underexpression of the gene *FGF1* (FDR = 1.2E‐31) in vascular cells, as *FGF1* has been reported to increase the expression of *NOTCH1* in vitro.^74^

Overall, these sex‐specific cell–cell communication patterns affecting multiple pathways across distinct cell types highlight complex and diverging intercellular signaling events occurring in male and female AD. The male‐specific overrepresentation of apoptosis‐related signaling changes contrasts with the female‐specific dominant changes associated with the Wnt signaling pathway and calcium signaling regulation. These disparities between the sexes underscore the importance of considering sex‐specific intercellular communication when studying AD pathogenesis.

### 3.4 GRN analysis

To gain a deeper understanding of the gene regulatory mechanisms associated with sex‐dependent DEGs and to identify key upstream regulators controlling these genes, we performed a GRN analysis for sex‐dependent DEGs (see section 2). Because astrocytes represent one of the largest cell type clusters in the scRNA‐seq data with the most pronounced sex‐dependent changes, we have focused on this cell type for this analysis, which requires large cell counts and substantial numbers of significant DEGs for robust GRN construction.

#### 3.4.1 Astrocytes: male‐specific changes

**FIGURE 4:**
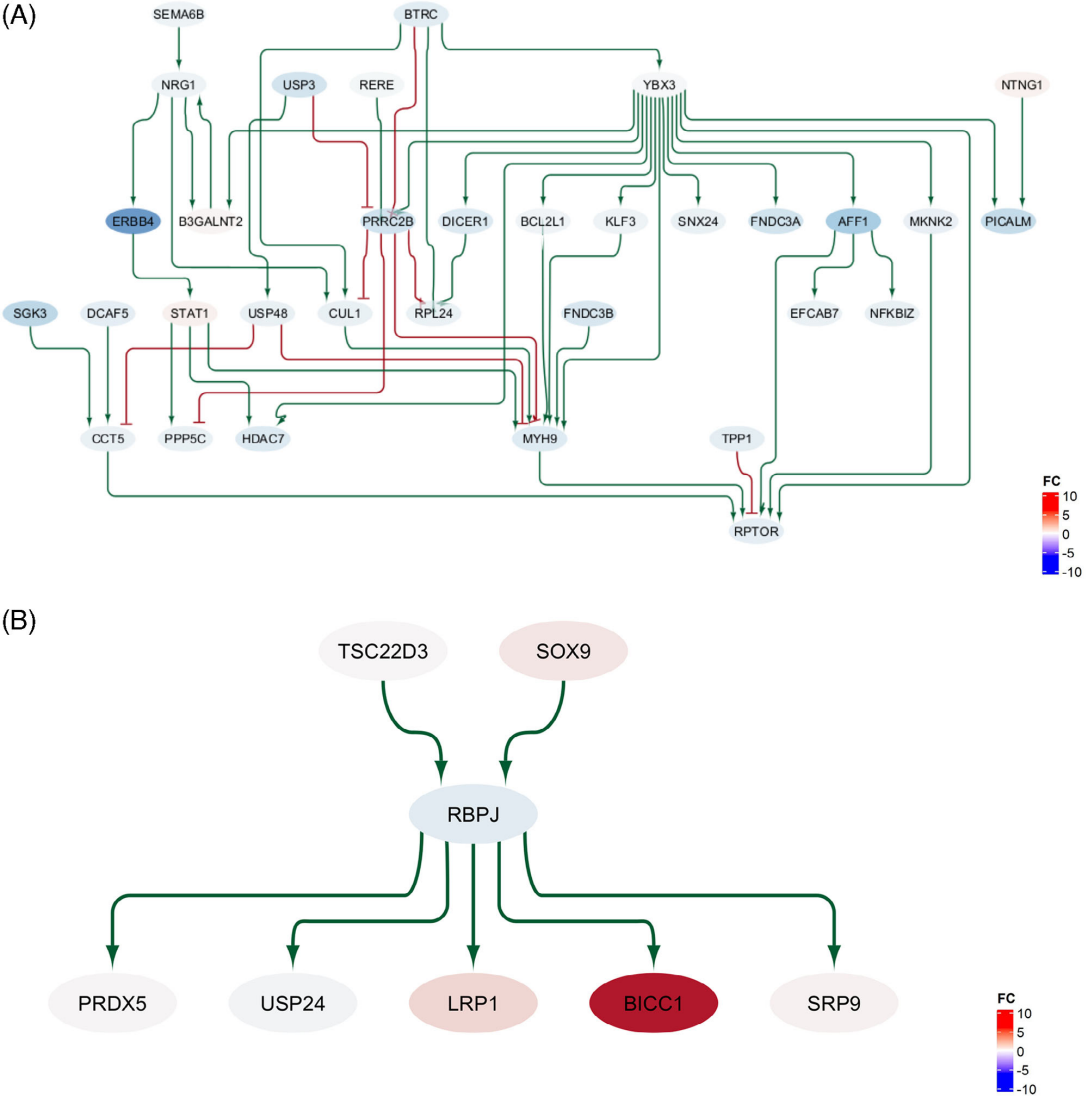
Graph representation of sub‐networks of the GRNs for male‐specific (A) and female‐specific (B) DEGs in astrocytes. As the complete networks are too large for interpretation, only the sub‐networks showing the upstream regulators of the key mediators *RPTOR* and *LRP1* are presented. In the graphs, green lines represent activating interactions and red lines represent inhibitory interactions. The node colors indicate the gene expression log fold change in the respective phenotype, transitioning from red (overexpressed) to blue (underexpressed), as shown in the color legend on the right. DEG, differentially expressed gene; GRN, gene regulatory network.

The network analysis of male‐specific DEGs in astrocytes (see Figure 4) revealed regulatory relationships between several of these genes in AD. Moreover, by performing a network perturbation analysis (see section 2), multiple genes were identified as important regulators in this network, with the ability to modulate the expression levels of many DEGs occurring downstream in the network (these genes are therefore termed “perturbagens” in the following sections).

The top‐ranked perturbagen was Neuregulin 1 (*NRG1*), with the ability to influence the expression of 64 downstream target genes, primarily through its interaction with *ERBB4*. *NRG1* mediates essential signaling pathways in neuronal survival, differentiation, and synaptic function. *NRG1–ERBB4* signaling has already been implicated in neurodevelopment and complex diseases, where they are involved in neuroprotective mechanisms and can prevent apoptosis under different toxic challenges.

Two further influential regulators identified are *CCT5* (chaperonin containing TCP1 subunit 5), which can modulate 61 downstream targets, and its upstream activator *SGK3* (serum/glucocorticoid‐regulated kinase 3). While *SGK3* is an estrogen‐inducible kinase that has been implicated in various survival pathways, the involvement of *CCT5* in this network may reflect its role as a chaperone in the chaperonin containing TCP1 complex (CCT). This complex has been implicated in neurodegenerative disorders involving defects in protein folding and aggregation. Finally, the gene *RPTOR* was identified as a key mediator of upstream changes in this network (see Figure 4A). *RPTOR* is a component of the mTORC1 complex, where it acts as a molecular adapter involved in many mTOR‐dependent signaling pathways, such as the regulation of cell growth and autophagy.

Together, the involvement of *NRG1*, *CCT5*, and *RPTOR* in male‐specific network alterations in AD suggests a potential interplay between sex‐dependent alterations in survival pathways, protein folding and aggregation processes, and mTOR signaling.

#### 3.4.2 Astrocytes: female‐specific changes

The GRN for female‐specific DEGs in astrocytes illustrates the complex regulatory interactions in this cell type in the context of AD pathology. Notably, the low‐density lipoprotein receptor‐related protein 1 (*LRP1*) was identified as a key mediator of expression changes in this network. *LRP1* is a TAU/MAPT receptor that plays an important role in modulating Aβ metabolism within the brain and peripheral tissues. It facilitates both the production of Aβ by promoting APP internalization and mediates the clearance of Aβ from the brain. Interestingly, the expression of this gene declines with age and has been found to be lower in frontal cortex tissue of AD patients than in controls.

The GRN reveals that the activation of *LRP1* is regulated through the transcription factor *RBPJ*, which is in turn activated by *SOX9* and *TSC22D3* (as depicted in Figure 4B). This regulatory cascade may be particularly relevant in the context of female‐specific changes in AD, as both *SOX9* and *TSC22D3* have been shown to be regulated by estrogen.

### 3.5 Comparison of sex‐dependent gene expression with protein expression data

To assess our transcriptomics findings at the protein level, we analyzed a previously published AD brain proteomics dataset^28^ (see section 2), focusing specifically on its overlap with the genes showing sex‐specific or sex‐dimorphic expression patterns in our scRNA‐seq analyses. While the bulk protein measurements cannot capture cell type–specific changes, this analysis identified several genes/proteins with consistent sex‐dependent changes at both transcript and protein level, providing robust evidence for sex‐specific molecular alterations in AD (see Table S8 in supporting information).

For the DEGs in astrocytes, we identified the overlapping calcium‐binding protein *S100A6* showing female‐specific changes, with increased expression at both transcript and protein levels in females (RNA FDR = 8.33E‐104, protein FDR = 0.037), while showing no significant changes in males. This calcium‐binding protein's altered expression suggests sex‐specific dysregulation of calcium homeostasis in astrocytes during AD progression.

In inhibitory neurons, *FAM160A2* showed female‐specific decreased expression at both the single‐cell transcript and bulk protein levels (RNA FDR = 7.84E‐29, protein FDR = 0.0024), with no significant changes in males. This gene's involvement in vesicle trafficking suggests sex‐specific alterations in cellular transport mechanisms.

In oligodendrocytes, we identified several male‐specific changes with consistent patterns across datasets. Notable examples include *ACIN1* (male RNA FDR = 6.80E‐159, protein FDR = 0.033), *CDC37* (male RNA FDR = 1.08E‐41, protein FDR = 0.026), and *PDK2* (male RNA FDR = 2.53E‐12, protein FDR = 0.014). These genes, involved in chromatin regulation (*ACIN1*), protein folding (*CDC37*), and metabolism (*PDK2*), suggest male‐specific alterations in nuclear processes and cellular metabolism.

We also identified several sex‐neutral genes showing significant changes in both sexes, including *PLEC* (astrocytes: male RNA FDR = 3.78E‐19, protein FDR = 0.032; female RNA FDR = 2.83E‐49, protein FDR = 2.43E‐04) and *SLC38A2* (astrocytes: male RNA FDR = 2.46E‐149, protein FDR = 0.036; female RNA FDR = 5.85E‐228, protein FDR = 0.006), indicating some disease processes affect both sexes similarly, though often with different magnitudes.

Overall, despite the different resolution of the two datasets, the validated sex‐dependent changes provide strong evidence for distinct molecular alterations in male versus female AD, particularly affecting calcium signaling, vesicular trafficking, nuclear processes, and cellular metabolism. The consistency between cell type–specific transcriptomic and bulk proteomic changes for these genes provides robust evidence for sex‐dependent molecular processes in AD and highlights potential sex‐specific biomarker signatures that could improve patient stratification.

### 3.6 Correlation between gene expression levels and age, Braak stage, and cognitive scores

To evaluate potential associations between the covariates age, Braak stage, and cognitive scores (using the Mini‐Mental State Examination [MMSE]) and the gene expression data, we created both general distribution plots and plotted the expression values of the main DEGs (see section 3.1) against cognitive scores (Figures S4 and S5 in supporting information), and assessed their Spearman correlations with age and Braak stage, stratified by sex (Table S9 in supporting information). We observed that multiple genes were significantly correlated with these covariates, matching with the fact that age is strongly associated with the risk of developing AD, and the association of AD with lower cognitive scores and detectable Braak pathology. Furthermore, matching with the sex‐dependent alterations observed for many genes in the differential expression analysis, some of the covariate correlations also displayed sex‐dependent patterns (see Table S9 for details). While the overall MMSE score distribution across the cohort showed the expected association with AD status (with MMSE scores < 20 for patients, and MMSE scores between 20 and 30 for controls), it did not differ significantly between the sexes (Figure S4). However, we observed sex‐dependent differences in the distribution of DEG expression levels across cognitive score ranges (Figure S5), in line with the sex dependencies in AD associations of these genes.

Apart from studying gene expression correlations with the covariates in general, we also specifically examined transcription factors that may influence our identified altered regulatory sub‐networks (see section 3.4), to check for potential correlations of their expression with age that could influence these sub‐networks (see Table S10 in supporting information). In astrocytes, we identified a significant correlation between the expression of the transcription factor *RBPJ* with age, but no other transcription factor with age‐dependent expression could be linked directly to the specific regulatory sub‐network alterations identified in our analyses.

## 4 DISCUSSION

This study used a comprehensive single‐cell transcriptomic atlas of the aged human prefrontal cortex to investigate sex‐dependent changes in AD at a systems level. Our analysis of key cell types, including astrocytes, neurons, immune cells, and vascular cells, revealed significant sex‐linked differences in gene expression, pathway alterations, and regulatory networks associated with AD pathology.

At the gene level, we identified numerous significant sex‐specific and sex‐dimorphic DEGs across cell types. In astrocytes, male‐specific changes were observed in genes such as *ERBB4*, involved in neuronal survival and synaptic plasticity, and *SGK3*, a regulator of cell growth and ion transport, while female‐specific changes included *CCL2* and *INHBA*, two important mediators of inflammation. These findings align with previous studies that have reported sex differences in AD risk, progression, and symptomatology.^49^ For instance, the male‐specific changes in *ERBB4* expression may contribute to the reported differences in synaptic plasticity and neuroinflammation between male and female AD patients.^30^, ^31^ Similarly, the female‐specific alterations in *CCL2* and *INHBA* expression in astrocytes could help explain the heightened inflammatory responses observed in female models of AD‐like pathology. Overall, these diverse gene‐level findings highlight the complex molecular landscape of AD and the importance of considering sex‐specific gene expression patterns in understanding the disease.

Pathway‐level analysis revealed further distinct changes between males and females at the global scale of cellular process activities. The enrichment of apoptotic and programmed cell death processes in male astrocytes, contrasted with alterations in Wnt signaling and cell‐cycle transition pathways in female astrocytes, suggests fundamentally different cellular responses to AD pathology between sexes. In excitatory neurons, males had enriched changes in particular in the regulation of apoptosis, whereas females showed alterations in calcium ion transport pathways. These findings may provide a molecular basis for the observed sex differences in neuronal loss and cognitive decline rates in AD.^8^ The male‐specific enrichment in apoptosis‐related pathways aligns with studies showing the activity of the pro‐apoptotic protein *BCL2*‐associated death promoter (BAD) is significantly elevated in neurons of male AD models, while the female‐specific alterations in Wnt signaling could contribute to the reported differences in synaptic plasticity and neurogenesis between sexes in AD.^69^, ^70^

In general, these sex‐dependent pathway changes highlight that in spite of many shared changes, there is also a broad divergence in several cellular responses to AD pathology between females and males.

GRN analyses provided further insight into sex‐specific regulatory mechanisms. In male astrocytes, *NRG1* and *CCT5* emerged as important regulators influencing the expression of many downstream target DEGs involved in cell survival and stress responses. In addition, *RPTOR* = was identified as a key mediator in this network. *RPTOR* is a critical component of the mTORC1 complex, which regulates cell growth, protein synthesis, and autophagy. The male‐specific changes in *RPTOR* suggest potential sex differences in mTOR signaling in AD, which could have implications for cellular metabolism and protein homeostasis. This finding is particularly intriguing as mTOR signaling has been implicated in the pathogenesis of AD by many studies and proposed as a therapeutic target.^40^, ^65^

In female astrocytes, the gene *LRP1* was identified as a central mediator of expression alterations in the network, with a known involvement in modulating Aβ metabolism. The coordinated expression changes observed in these cell type–specific regulatory networks point to sex‐specific regulatory hubs that may warrant further study as potential drivers of sex‐dependent pathological processes in AD. *NRG1* has previously already been proposed as a therapeutic target for AD, but the results obtained here indicate that its therapeutic potential may be sex dependent. Similarly, the identification of *RPTOR* as a male‐specific regulator suggests that targeting mTOR signaling in AD might require sex‐specific approaches.

Our analysis of cell–cell communication revealed distinct patterns of intercellular signaling in male and female AD. A male‐specific impact of changes on apoptosis‐related signaling contrasted with a female‐specific focus on Wnt pathway and calcium signal regulation. These differences in intercellular communication indicate that sex‐specific alterations can also affect how different cell types interact and contribute to AD pathology.

Finally, by integrating complementary proteomic data, we were able to confirm multiple transcriptomics findings at the protein level. However, we also acknowledge certain limitations in this validation approach: proteomic data only provides incomplete coverage of the full proteome, and protein translation rates do not always directly correspond to transcription rate changes, resulting in different changes observed at the mRNA and protein levels. Consequently, we were not able to confirm matching results for all genes of interest, but the overlapping gene‐ and protein‐level findings provide a starting point for further follow‐up analyses.

Taken together, these findings underscore the importance of considering sex as a biological variable in AD research and in the development of targeted therapies. The identification of sex‐specific molecular targets and cellular processes can provide a basis for future studies aimed at developing more tailored therapeutic approaches. However, it is important to note that further research is needed to fully understand the mechanisms and functional implications of these molecular differences and their potential for translation into clinical applications. In particular, future research directions could include functional validation of key sex‐specific genes and pathways using in vitro and in vivo models, investigation of the temporal dynamics of these sex‐specific changes at different stages of AD progression, exploration of potential sex‐specific biomarkers for early detection and monitoring of AD, and development and testing of sex‐specific and sex‐adjusted therapeutic interventions targeting the pathways and networks identified in this study.

In conclusion, our study highlights the complex and multifaceted nature of sex‐dependent molecular changes in AD. Continued research efforts in this direction may contribute to more personalized and effective approaches to the diagnosis, treatment, and potential prevention of AD in both male and female patients. By recognizing and further investigating these sex‐dependent aspects of AD pathology, we can move closer to a more comprehensive understanding of AD mechanisms and potential avenues for more personalized intervention.

## CONFLICT OF INTEREST STATEMENT

The authors have declared that no competing interests exist. Author disclosures are available in the supporting information.

## CONSENT STATEMENT

All data analyzed were obtained from previous studies in which all human subjects provided informed consent.

## CODE AVAILABILITY

The code for this study is available at https://gitlab.lcsb.uni.lu/bds/ad‐sex‐specific‐differences.

## Supporting information



Supporting Information

Supporting Information

Supporting Information

Supporting Information

Supporting Information

Supporting Information

Supporting Information

Supporting Information

Supporting Information

Supporting Information

Supporting Information

Supporting Information

## Data Availability

The ROSMAP data and metadata are available from the Synapse AD Knowledge Portal (https://www.synapse.org/#!Synapse:syn52293417).
