# Supplementary material for: Sex‐dependent molecular landscape of Alzheimer's disease revealed by large‐scale single‐cell transcriptomics
Source: Alzheimers Dement. 2024 Dec 31;21(2):e14476. doi: 10.1002/alz.14476 (PMC11848167; doi:10.1002/alz.14476)
Supplement: Supplementary file 1 — Supporting Information [file ALZ-21-e14476-s012.pdf]

Supplementary Figures to
"Sex-Dependent Molecular Landscape of Alzheimer's Disease Revealed by Large-Scale Single-Cell Transcriptomics"

Mohamed Soudy [a], Sophie Le Bars [a], Enrico Glaab [a*]

[a] *Biomedical Data Science, Luxembourg Centre for Systems Biomedicine (LCSB), University of Luxembourg, Esch-sur-Alzette, Luxembourg*

[*] *Contact: enrico.glaab@uni.lu*

Supplementary Figures - Overview

1. Expression of genes with sex-dependent alterations in AD across Braak stages....................... 2
2. Female-specific pathway enrichment analysis (Astrocytes) 3
3. Female-specific cell-cell communication (Astrocytes) 4
4. Distribution of Braak stages across the samples in the cohort 5
5. Gene expression vs. Mini-Mental State Examination (MMSE) scores..................................... 6

1. Expression of genes with sex-dependent alterations in AD across Braak stages

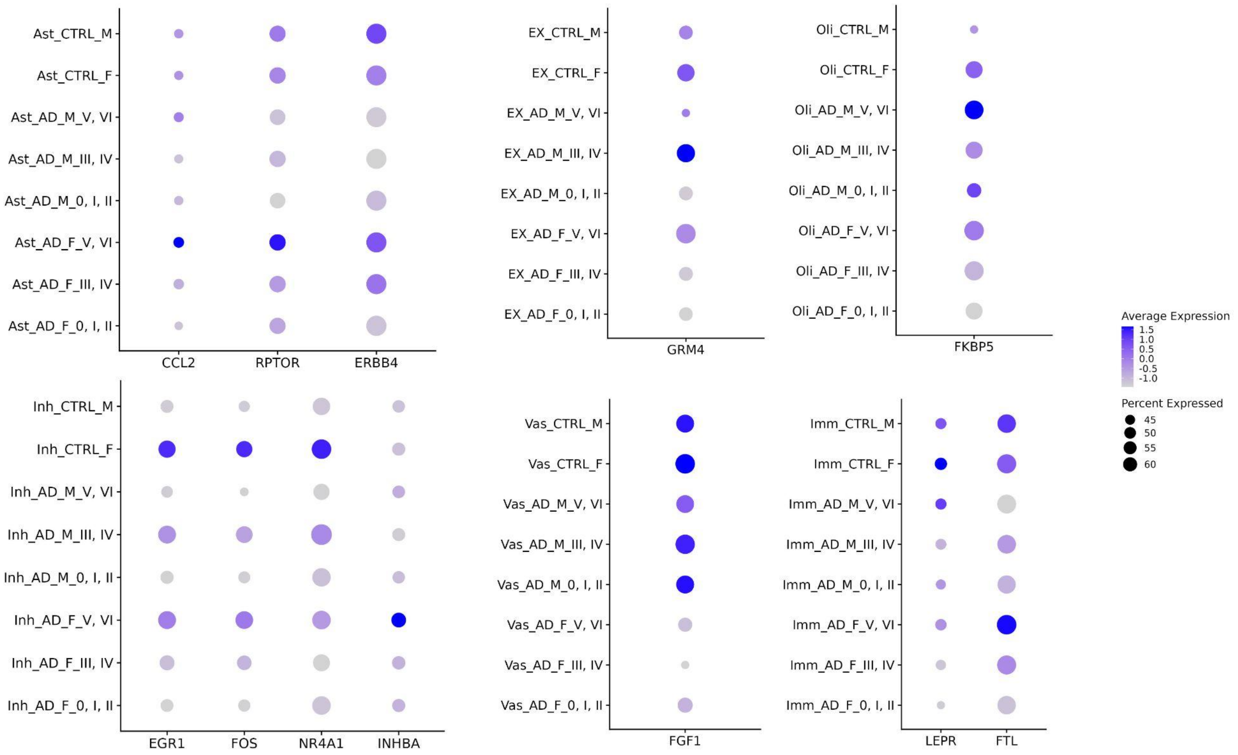

Supplementary Figure 1: Dot plots showing gene expression patterns for key genes with sex-dependent differential expression across diverse cell populations in Alzheimer's disease (AD) and control samples, stratified by sex and Braak stages (see the main manuscript for a detailed discussion of these gene). Each dot's diameter indicates the percentage of cells expressing the gene, while the color intensity (gray to dark purple) represents the average expression level. The visualization encompasses six major cell types (vertical axis): Astrocytes (Ast), Excitatory neurons (EX), Inhibitory neurons (Inh), Oligodendrocytes (Oli), Vascular cells (Vas), and Immune cells (Imm). Samples are categorized by disease status (AD vs CTRL), sex (F: female, M: male), and Braak stages (0-II, III-IV, V-VI), reflecting the progressive spread of AD pathology from the medulla oblongata through limbic structures to the neocortex. Key genes analyzed include markers for inflammation (*CCL2*), metabolism (*RPTOR*, *ERBB4*), cellular signaling (*EGR1*, *FOS*, *NR4A1*, *INHBA*), neurotransmission (*GRM4*), lipid processing (*FXBP5*), vascular function (*FGF1*), and immune response (*LEPR*, *FTL*). This comprehensive analysis reveals cell-type-specific and sex-dependent expression patterns across the different Braak stages that may contribute to AD pathogenesis and progression.

2. Female-specific pathway enrichment analysis (Astrocytes)

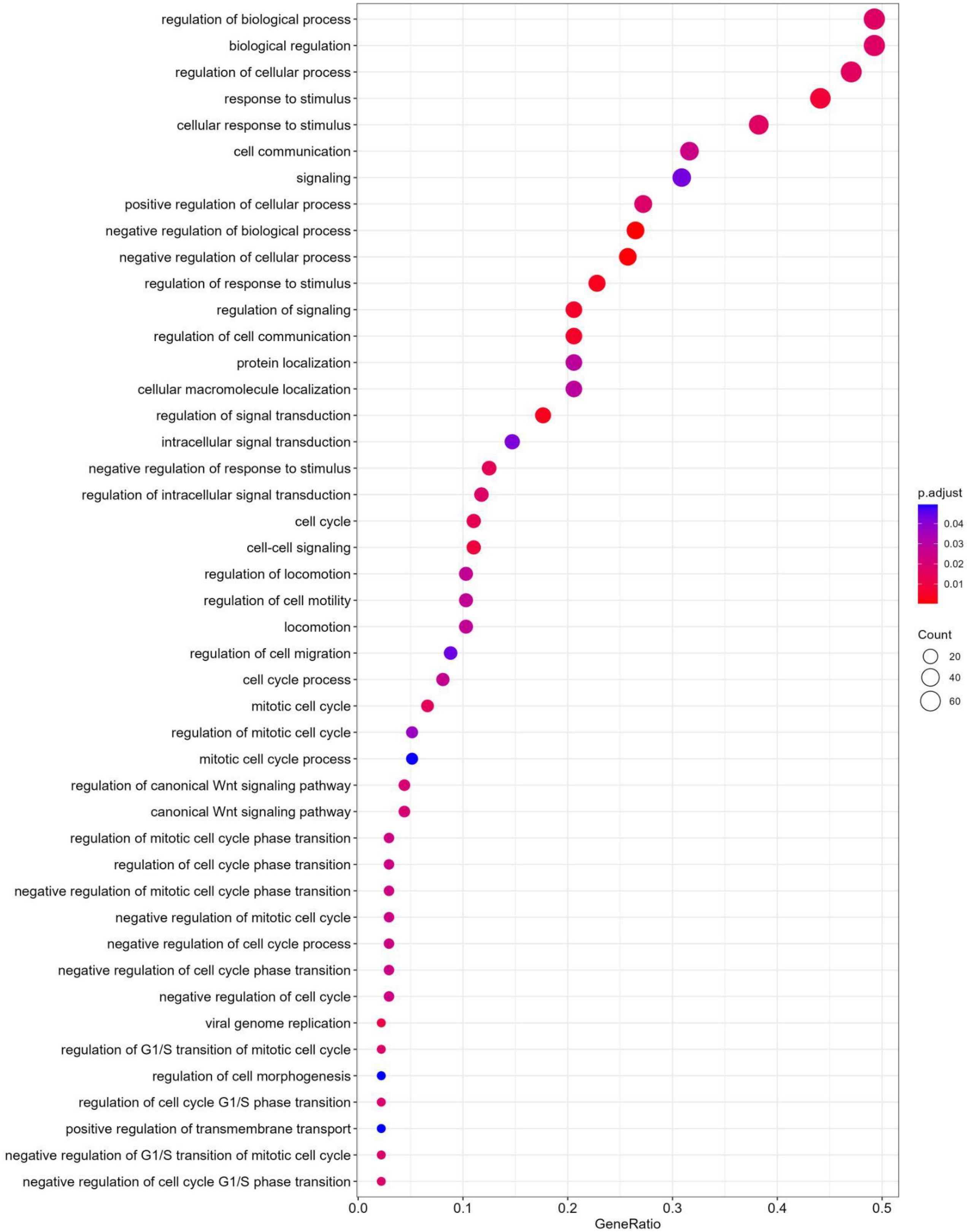

Supplementary Figure 2: Dot plot visualization of the female-specific Gene Ontology (GO)

enrichment analysis highlighting biological processes uniquely altered in female astrocytes in Alzheimer's disease (AD) vs. controls. The analysis reveals GO terms that are significantly enriched (adjusted $p < 0.05$) in female-specific differentially expressed genes (DEGs) but that show no significant association even at nominal level ($p > 0.1$) in male AD astrocytes. The x-axis represents the GeneRatio (proportion of DEGs associated with each GO term), while the y-axis lists the enriched biological processes, arranged by significance. Dot size indicates the count of genes associated with each term, and color intensity (red to purple) reflects statistical significance. Notable enriched processes include broad cellular regulatory mechanisms (biological regulation, regulation of cellular processes), stimulus response pathways, cell signaling cascades, and specific cell cycle-related processes, suggesting distinct sex-specific molecular alterations in astrocytes during AD pathogenesis.

3. Female-specific cell-cell communication (Astrocytes)

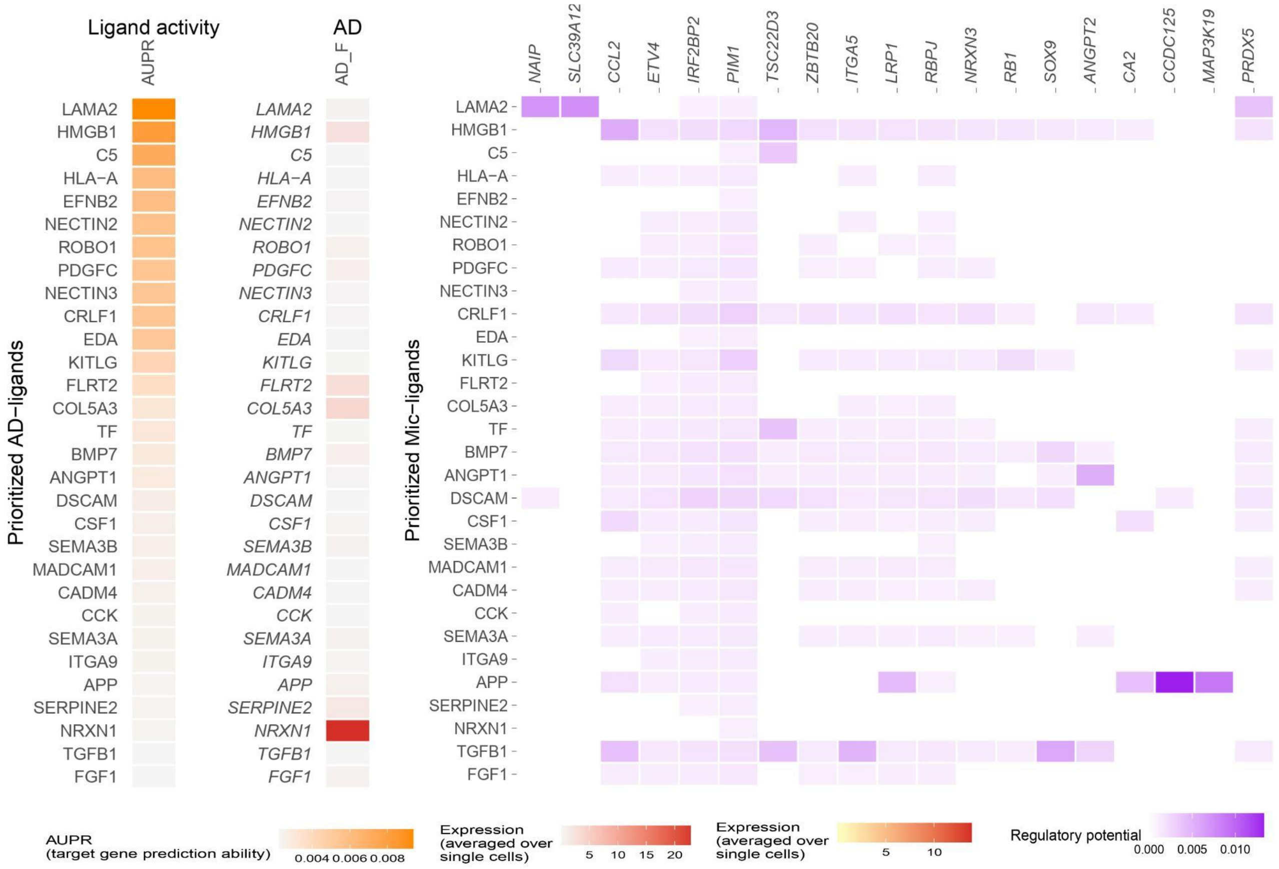

Supplementary Figure 3: Comprehensive analysis of cell-cell communication signatures in female astrocytes in Alzheimer's disease (AD) vs. controls. The figure is organized into three panels: (Left) AUPR scores indicating the ligand activity/target gene prediction ability (orange color scale); (Center) AD-specific ligand expression patterns (red intensity); (Right) Regulatory potential of downstream target genes across different cell types (purple intensity). The y-axis lists the top 30 prioritized ligands involved in astrocyte-mediated cellular communication, while the x-axis in the rightmost panel shows their target genes. Notable signaling molecules include key immune regulators (*HLA-A*), growth factors (*TGFB1*, *FGF1*), cell adhesion molecules (*NECTIN2*, *ROBO1*), and neuroinflammatory mediators

(*SERPINE2*, *CCK*). The color intensity scales represent AUPR scores (0.004-0.006), expression levels (5-15), and regulatory potential (0.00-0.015) respectively. This analysis highlights the complex interplay of signaling pathways and molecular interactions in astrocytes in the female AD brain, highlighting potential key signaling molecules with sex-specific patterns.

4. Distribution of Braak stages across the samples in the cohort

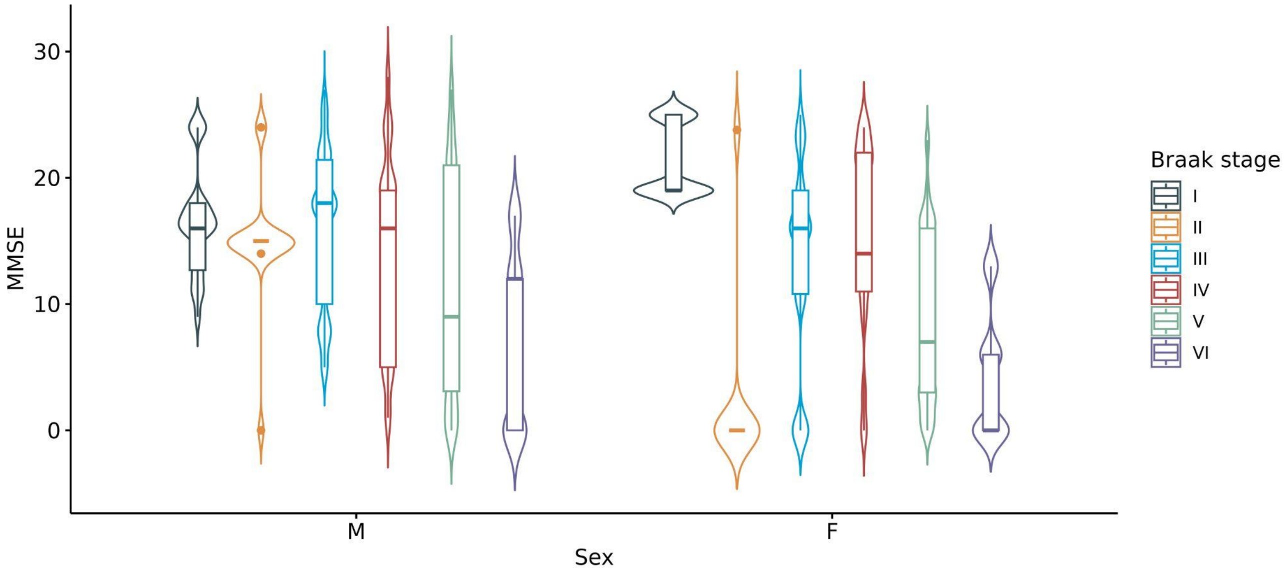

Supplementary Figure 4: Distribution of cognitive scores (measured using the Mini-Mental State Examination, MMSE) in the cohort, stratified by sex and Braak stage (I – VI). Each violin plot represents the range and density of MMSE scores within each Braak stage for males (M) and females (F). Box plots within the violins show the interquartile range and median scores.

5. Gene expression vs. Mini-Mental State Examination (MMSE) scores

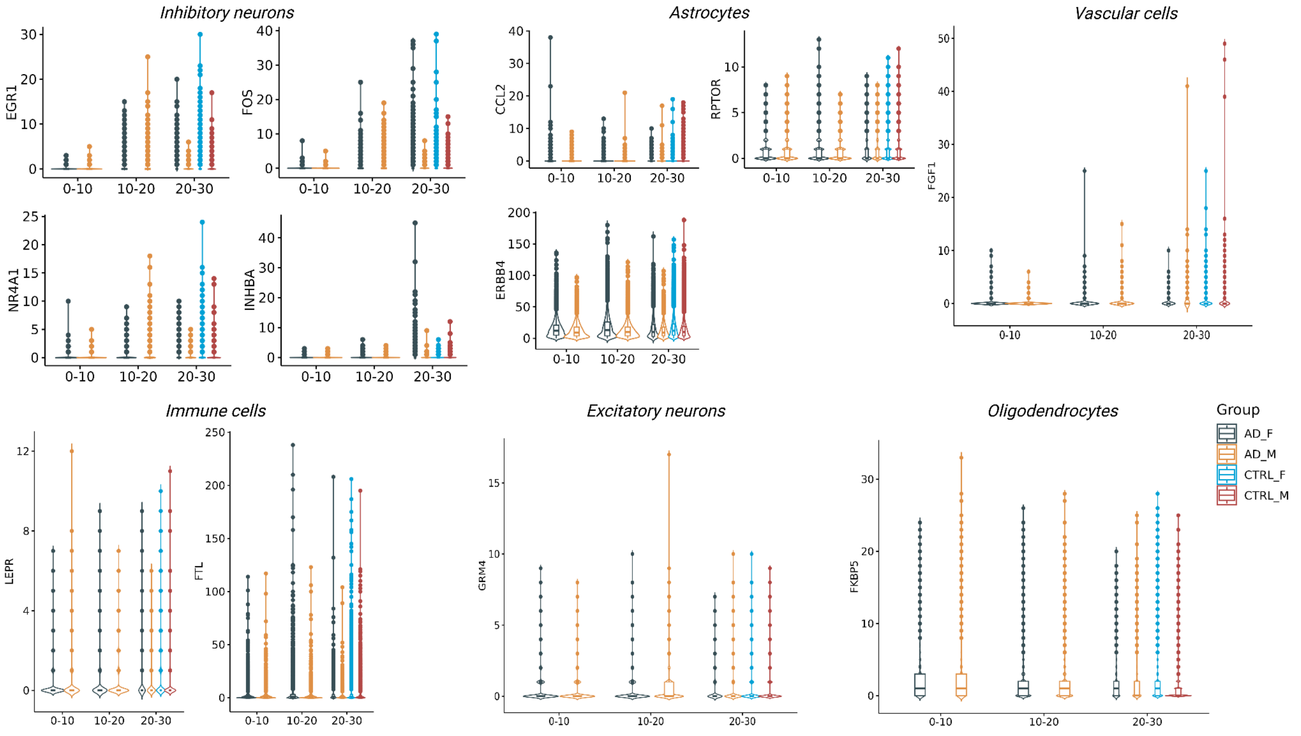

Supplementary Figure 5: Plots of the expression levels for key genes with sex-dependent alterations in AD (see the discussion of these gene in the main manuscript) versus cognitive scores (measured using the Mini-Mental State Examination, MMSE), separated by sex and condition. Each panel represents a specific gene and cell type, with groups denoted by Alzheimer's disease females (AD_F), Alzheimer's disease males (AD_M), control females (CTRL_F), and control males (CTRL_M). These plots illustrate significant differences in the distribution of expression levels across cognitive score ranges.
