# Supplementary material for: Sex‐dependent molecular landscape of Alzheimer's disease revealed by large‐scale single‐cell transcriptomics
Source: Alzheimers Dement. 2024 Dec 31;21(2):e14476. doi: 10.1002/alz.14476 (PMC11848167; doi:10.1002/alz.14476)
Supplement: Supplementary file 2 — Supporting Information [file ALZ-21-e14476-s011.docx]

| **Cell type** | **Male-specific DEGs** | **Female-specific DEGs** | **Sex-shared DEGs** | **Sex-dimorphic DEGs** |
| --- | --- | --- | --- | --- |
| **Astrocytes** | 208 | 136 | 744 | 502 |
| **Excitatory neurons, subgroup 1** | 129 | 533 | 1126 | 1351 |
| **Excitatory neurons, subgroup 2** | 87 | 1080 | 1982 | 2504 |
| **Excitatory neurons, subgroup 3** | 122 | 147 | 3091 | 3515 |
| **Immune cells** | 56 | 37 | 29 | 5 |
| **Inhibitory neurons** | 15 | 119 | 14 | 5 |
| **Oligodendrocytes** | 834 | 50 | 687 | 781 |
| **Oligodendrocyte precursor cells** | 45 | 14 | 5 | 6 |
| **Vasculature cells** | 104 | 34 | 10 | 24 |

**Suppl. Table 1**: Overview of the number of sex-dependent DEGs for each cell type. The first column lists the cell types, and the following columns contain the number of male-specific, female-specific, and sex-dimorphic DEGs, respectively. DEGs have been determined using an FDR-adjusted p-value threshold of 0.05.
