# Supplementary material for: Sex‐dependent molecular landscape of Alzheimer's disease revealed by large‐scale single‐cell transcriptomics
Source: Alzheimers Dement. 2024 Dec 31;21(2):e14476. doi: 10.1002/alz.14476 (PMC11848167; doi:10.1002/alz.14476)
Supplement: Supplementary file 7 — Supporting Information [file ALZ-21-e14476-s009.docx]

|  | **Cell Type** | **ID** | **Pathway** | **P-adjusted** |
| --- | --- | --- | --- | --- |
| **Male-**  **Specific** | Astrocytes | GO:0097048 | Dendritic cell apoptotic process | 0.01 |
|  |  | GO:0060561 | Apoptotic process involved in morphogenesis | 0.001 |
|  |  | GO:0071636 | Positive regulation of transforming growth factor beta production | 0.016 |
|  |  | GO:0036303 | Lymph vessel morphogenesis | 0.016 |
|  | Excitatory neurons | GO:0043069 | Negative regulation of programmed cell death | 0.005 |
|  |  | GO:0043069 | Response to cytokine | 0.005 |
|  | Vascular cells | GO:0033028 | Myeloid cell apoptotic process | 0.001 |
|  |  | GO:0033033 | Negative regulation of myeloid cell apoptotic process | 0.0026 |
|  |  | GO:0007213 | G protein-coupled acetylcholine receptor signaling pathway | 0.015 |
|  |  | GO:0099558 | Maintenance of synapse structure | 0.015 |
| **Female-**  **Specific** | Astrocytes | GO:0046579 | Positive regulation of Ras protein signal transduction | 0.001 |
|  |  | GO:0090263 | Positive regulation of canonical Wnt signaling pathway | 0.014 |
|  |  | GO:0007254 | JNK cascade | 0.025 |
|  |  | GO:0022407 | Regulation of cell-cell adhesion | 0.015 |
|  | Excitatory neurons | GO:0010522 | Regulation of calcium ion transport into cytosol | 0.014 |
|  |  | GO:0050926 | Regulation of positive chemotaxis | 0.007 |
|  | Vascular cells | GO:0050808 | Synapse organization | 1.63e-11 |
|  |  | GO:0007219 | Notch signaling pathway | 2.76e-06 |

**Supplementary Table 6**: Male and female-specific significant Gene Ontology terms in each cell type with the highest significance (FDR < 0.05, up to 10 terms per cell type shown), i.e., representing the terms significant in only one sex (FDR < 0.05) which do not approach significance in the other sex (p > 0.1).
